# Upregulating endogenous genes by an RNA-programmable artificial transactivator

**DOI:** 10.1093/nar/gkv682

**Published:** 2015-07-07

**Authors:** Cristina Fimiani, Elisa Goina, Antonello Mallamaci

**Affiliations:** Laboratory of Cerebral Cortex Development, SISSA, Trieste, 34136, Italy

## Abstract

To promote expression of endogenous genes *ad libitum*, we developed a novel, programmable transcription factor prototype. Kept together via an MS2 coat protein/RNA interface, it includes a fixed, polypeptidic transactivating domain and a variable RNA domain that recognizes the desired gene. Thanks to this device, we specifically upregulated five genes, in cell lines and primary cultures of murine pallial precursors. Gene upregulation was small, however sufficient to robustly inhibit neuronal differentiation. The transactivator interacted with target gene chromatin via its RNA cofactor. Its activity was restricted to cells in which the target gene is normally transcribed. Our device might be useful for specific applications. However for this purpose, it will require an improvement of its transactivation power as well as a better characterization of its target specificity and mechanism of action.

## INTRODUCTION

Artificial transactivation of specific endogenous genes *ad libitum* is a desirable goal for a number of basic and applied research purposes. This goal has been achieved by short siRNA/miRNA-like molecules targeting gene promoters (1–4), termini (5) or enhancers (6). Commonly referred to as RNA activation, this procedure has been successfully implemented for a large number of genes (7). Its effectors are expected to destabilize transcription-inhibiting ncRNAs or ease the recruitment of transcription-promoting complexes to chromatin (reviewed in (8)). Alternatively, transactivation of endogenous genes may be obtained by dedicated artificial enzymes, able to recognize arbitrary target sequences by polypeptidic, Zinc finger- (ZF-) type (9–13) and TransActivator Like Element- (TALE-) type (14–16), DNA-interacting domains. Albeit nicely working, these enzymes may display suboptimal efficiency and/or specificity of DNA–protein interaction. Large sizes of their DNA-binding domains may, as well, pose problems of cDNA synthesis and delivery (17). Recently, the allocation of the DNA-recognition function to a guide-RNA cofactor, as implemented in artificial, Clustered Regularly Interspaced Short Palindromic Repeat (CRISPR)-derived transactivators (18–23), allowed to solve DNA recognition problems thanks to a far simplified and straightforward approach. However, CRISPR molecules are even larger than TALE- and ZF-transactivators (24).

To address these issues, we have conceived a novel, small and non-CRISPR-based device. This consists of a fully synthetic, ribonucleoprotein transcription factor, including a polypeptidic trans-activating domain as well as a non-coding RNA ‘bait’ domain. The former stimulates transcription. The latter specifically drives the whole ribonucleoprotein to the target gene. The two elements are kept together by two ancillary domains, a polypeptidic MS2 RNA-interacting domain (25), covalently joined to the former and forming with it the polypeptidic ‘apo-factor’, and its corresponding hairpin RNA interactor (26,27), covalently joined to the latter and forming with it the RNA ‘cofactor’.

As a proof of principle, we assessed the feasibility of this design with three genes highly expressed in HEK293T cells, *FMR1*, *NAP22* and *NRGN*. Moreover, we employed this device to stimulate two transcription factor genes involved in early cortico-cerebral development, *Emx2* and *Foxg1*. These genes control pallial field specification (28,29), dictate precursors population kinetics (6,30–34) and modulate laminar (35,36) as well as areal (37,38) neuronal differentiation. We selected them, because of high responsivity of proliferating pallial precursors to even small changes of their expression levels (6,39–41), offering an easily detectable biological readout of the efficacy of our procedure, as well as because of potential therapeutic interest of their artificial manipulation (34).

Remarkably, our device upregulated all five selected genes. Gene upregulation was small, however specific. In case of cortico-cerebral precursors, it led to a clear reduction of neuronal differentiation. Notably, the activity of our device was confined to cells already expressing the gene of interest (GOI). Finally, performances of our transactivator could be optimized, by improving key features of its apofactor and cofactor components.

## MATERIALS AND METHODS

### Animal handling

Wild-type, CD1 strain mice used in this study were purchased from Harlan-Italy and housed at the SISSA mouse facility. Embryos were staged by timed breeding and vaginal plug inspection. Animals handling and subsequent procedures were in accordance with European laws [European Communities Council Directive of November 24, 1986 (86/609/EEC)] and with the National Institutes of Health guidelines. Embryos (E10.5 and E12.5) were harvested from pregnant dames killed by cervical dislocation and put in sterile ice-cold PBS supplemented with 0.6% glucose. Cerebral cortices (E12.5), mesencephalons (E10.5) and rhombo-spinal tracts (E10.5) were then dissected and collected in the same solution.

### Cell culture

Embryonic cortico-cerebral tissue was mechanically dissociated to single cells by gentle pipetting. Neural precursor cells were subsequently counted in a Burker chamber and plated in 24-multiwell plates (Falcon), at the density of 1000 cells/μl, in a proliferative medium (DMEM-F12, 1X Glutamax (Gibco), 1X N2 (Invitrogen), 1 mg/ml BSA, 0.6% glucose, 2 μg/ml heparin (Stem Cell Technologies), 20 ng/ml bFGF (Invitrogen), 20 ng/ml EGF (Invitrogen), 1X Pen-Strept (Gibco), 10 pg/ml Fungizone (Gibco)). Neural precursors were acutely infected by recombinant lentiviruses and kept in culture up to 96 h.

HEK293T cells were cultured in Iscove's Modified Dulbecco's Medium 1X (Gibco) supplemented with 10% FBS (Sigma). They were used for lentiviral production and titration as well as for *FMR1*, *NAP22* and *NRGR* experiments.

### Building the NMHV constructs

The codon- and restriction enzyme-optimized coding sequence (cds) of NMHV (Nuclear-localization-signal, multimerized-MS2 peptide, Hemagglutinin-antigen, Virion-peptide-16) apo-transactivator is the following:

ACCATGGTTCCAAAAAAGAAGAGAAAGGTGCCAAAAAAGAAGAGAAAGGTA GCTTCTAACTTTACTCAGTTCGTTCTCGTGGAAAATGGCGGAACTGGCGAC GTGACTGTCGCCCCAAGCAACTTCGCTAACGGGGTCGCTGAATGGATCAGC TCTAACTCGCGTTCACAGGCTTACAAAGTAACCTGTAGCGTTCGTCAGAGC TCTGCGCAGAATCGCAAATACACCATCAAAGTCGAGGTGCCTAAAGTGGCA ACCCAGACTGTTGGTGGAGAGGAGCTTCCTGTAGCCGGCTGGCGTTCGTAC TTAAATATGGAACTAACCATTCCAATTTTCGCTACGAATTCCGACTGCGAG CTTATTGTTAAGGCAATGCAAGGTCTCCTAAAAGATGGAAACCCGATTCCC TCAGCAATCGCAGCAAACTCCGGCATCTACTATCCGTATGATGTGCCGGAT TATGCGACCGGTGACGCCCTTGACGATTTTGACTTAGACATGCTCCCAGCC GATGCCCTTGACGACTTTGACCTTGATATGCTGCCTGCTGACGCTCTTGAC GATTTTGACCTTGACATGCTCCCAGGCTAA (here, Kozak/start, NLS(2x), MS2_coat potein, HA_tag, AgeI restriction site, VP16_transactivating peptide (3x) and stop modules are highlighted by alternate styles, normal and underlined).

NMHV cds was synthesized, cloned into pUC57, grown in Xl1-blue cells and sequence-verified for us by GenScript. The cds was transferred as BamHI/SalI fragment into BamHI-SalI cut pCCLsin.PPT.hPGK.EGFP.Wpre (42). The resulting plasmid, LV_NMHV, was subsequently grown in ElectroMAX^TM^ Stbl 4^TM^ (Invitrogen) cells and sequence-verified. LV_NMHV was used as an expression plasmid for *FMR1*, *NAP22* and *NRGN* experiments in HEK293T cells. The same construct was utilized in producing the corresponding recombinant lentivirus, for *Emx2* and *Foxg1* assays in neural precursors.

Please note that pCCLsin.PPT.hPGK. EGFP.Wpre was used as the negative control for LV_NMHV and is referred to as ‘EGFP’, unless otherwise indicated.

The NMHE expressing lentivector was obtained from LV_NMHV, replacing its AgeI/SalI fragment (encoding for the VP16 peptide) by the 141bp AgeI/SalI fragment ACCGGTTACCTGAGCACCCAGTCCGCCCTGAGCAAAGACCCCAACGAGAAG CGCGATCACATGGTCCTGCTGGAGTTCGTGACCGCCGCCGGGATCACTCTC GGCATGGACGAGCTGTACAAGTAAAGCGGCCGCGTCGAC (encoding for the carboxyterminal EGFP peptide YLSTQSALSKDPNEKRDHMVLLEFVTAAGITLGMDELYK).

### Building ncRNA constructs

The sequence of the RNA cofactor fragment MF6 was as follows (‘MS2 coat protein-binding stem-and-loop moieties’ are underlined):

MF6_AGATCTCGGGAAACATGAGGATCACCCATGTCG CCCGCTCCACCCAAACAACCCCCTAAACATGAGGATCACCCATGTCG AGGGCACCACCCAAACAAACAATGAAACATGAGGATCACCCATGTCG CATTCTCCAACCAACCAACCGGGGAAACATGAGGATCACCCATGTCG CCCCCTACACCCAAACAACCGCGCAAACATGAGGATCACCCATGTCG CGCGCATCACCCAAACAAACCAGGAAACATGAGGATCACCCATGTCG CCTGGGATCCACCGATATC

MF6 was cloned as BglII/EcoRV fragment into BamHI-EcoRV cut pcDNA3ΔPvuII, thus obtaining MF6-ø.

*FMR1-*, *NAP22-* and *NRGN-*specific, wild-type ncRNA baits were amplified from genomic DNA by the oligos reported in Supplementary Table S1. NAP22.60L-derivative, mutant baits (for sequences, see Supplementary Table S7) were chemically synthesized as dsDNAs, flanked by sticky, BamHI/SalI compatible adaptors. All baits were then cloned as BamHI/XhoI or BamHI/SalI fragments into the BamHI/XhoI cut MF6-øplasmid, thus obtaining the corresponding MF6-bait constructs.

Sequences of the RNA cofactor fragments MF1 and MF2 were as follows (here ‘MS2 coat protein-binding stem-and-loop moieties’ are underlined):

MF1_GATCGATATCCGGGAAACATGAGGATCACCCATGTCGCCCGCAGCGGATCCCCCGTCGACTTT TTTGGTACC;

MF2_GATCGATATCCGGGAAACATGAGGATCACCCATGTCGCCCGCAGCGGATCGATATCCGGGAAACATGAGGATCACCCATGTCGCCCGCAGCGGAT CCCCCGTCGACTTTTTTGGTACC.

MF1 was cloned as BamHI-compatible/KpnI fragment into BamHI-KpnI cut pCCLsin.PPT.hPGK.EGFP.Wpre vector (42), thus obtaining LV_MF1-ø. LV_MF2-øwas obtained by cloning an additional MF1 finger (a BamHI-compatible/KpnI fragment) into BamHI/KpnI cut LV_MF1-ø.

The *Emx2* and *FoxG1* ncRNA baits were generally amplified from genomic DNA, by the oligos reported in Supplementary Table S2. The *Emx2* 30bp bait was obtained by annealing the two oligos reported in the same table. Baits were cloned as BglII/XhoI fragments into BamHI/SalI cut LV_MF1/2-øplasmids, thus obtaining the corresponding LV_MF1-bait and LV_MF2-bait constructs.

### Building accessory expression plasmids

Plasmids driving constitutive expression of mCherry and EGFP referred to in Supplementary Figures S1 and S5, Pgkp1-mCherry and CMVp-EGFP, were obtained by replacing EGFP cds by mCherry cds in pCCLsin.PPT.hPGK.EGFP.Wpre and by cloning EGFP cds into pcDNA3ΔPvuII, respectively.

### HEK293T cell cotransfection

Cells were generally cotransfected by LipoD reagent, according to Manufacturer's instructions. Where not otherwise specified, aliquots of 10^6^ cells were cotransfected by 0.75 μg of a ncRNA cofactor-expressing plasmid, 1.25 μg of an apofactor (or a control)-expressing plasmid and 0.25 μg of pcDNA3ΔPvuII-EGFP (see above), as internal control.

In specific cases (as highlighted in legends to figures), cells were cotransfected by Lipofectamine 3000 reagent, according to manufacturer's instructions. Here two rounds of cotransfection were performed, at a 12-hour interval. In each round, where not otherwise specified, aliquots of 10^6^ cells were cotransfected by 0.95 μg of ncRNA cofactor-expressing plasmid and 1.60 μg of apofactor (or control)-expressing plasmid.

### Recombinant lentivirus production

Recombinant third generation self-inactivating (SIN) lentiviruses were produced and titrated as previously described (34).

### Off-target gene selection

ncRNA bait sequences were blasted against human and murine genomes by Blast software (accessible at http://blast.ncbi.nlm.nih.gov and run online with the following parameters: blastn, ‘for somewhat similar sequences’; expect threshold: 40; word size: 7; max matches in a query range : 0; match/mismatch scores, 2, -3; gap costs: existence 2, extension 2; filter low complexity regions: no; filter species-specific repeats: no). Homologous modules found by Blast were then prioritized and selected, based on their length (between 50 and 110 bp), gap density (<20%) and identity (>70%). Selected modules were subsequently mapped to the transcriptome, using the UCSC Genome browser (accessible at http://genome.ucsc.edu; assemblies mm10 and hg19) and the Ensemble-GENCODE track (accessible at http://www.ensembl.org). Lastly, they were filtered on the basis of their distance from transcriptional start-site (TSS) of potential off-target genes (from −0.5 kb to +0.5 kb). The results are summarized in Supplementary Tables S5 and S6.

### RNA profiling

Total RNA was extracted from cells using TRIzol Reagent (Invitrogen) according to manufacturer's instructions. Agarose gel electrophoresis and spectrophotometric measurements (NanoDrop ND-1000) were employed to estimate its concentration, quality and purity.

At least 0.5 μg of total RNA from each sample was retrotranscribed by SuperScriptIII^TM^ (Invitrogen) in the presence of random hexamers, according to the manufacturer's instructions. 1/100 of the resulting cDNA was used as substrate of any subsequent qPCR reaction. Limited to the intronless amplicons, prior to the retrotranscription, RNA preparations were treated by DNAseI (2U/μg of RNA) 1 h at 37°C, and processed by RNeasy Mini Kit (Qiagen). Next, negative control PCRs were run on RT^−^ cDNA preparations. In general, PCR reactions were performed by the SsoAdvanced SYBR Green Supermix^TM^ platform (Biorad), according to manufacturer's instructions. For each transcript under examination and each sample, cDNA was PCR-analyzed in technical triplicate, against absolute standards, and average results calculated. Averages were normalized against *Gapdh* and further normalized against controls. Experiments were performed at least in biological triplicate and analyzed by Student's t-test. Oligos were as in Supplementary Table S3.

### ChIP-qPCR

The chromatin immunoprecipitation quantitative polymerase chain reaction assays (ChIP-qPCRs) were performed on chromatin extracted from HEK293T cells (case *NAP22*) or neural cell cultures (case *Emx2*). Cells were transfected with constitutive expression plasmids (case *NAP22*) or acutely infected with bio-active and control lentiviruses (case *Emx2*), similar to transactivation assays. Then, they were kept in culture for 72 and 96 h, respectively. ChIP analysis was performed according to the MAGnify™ Chromatin Immunoprecipitation System protocol (Invitrogen), with minor modifications.

For each ChIP assay, chromatin from 10^6^ cells was fixed by 1% formaldehyde, for 10 min at RT. After cell lysis, fixed chromatin was sonicated by a Soniprep 150 apparatus (on ice; 5 s ON, 55 s OFF; oscillation amplitude five microns; four cycles) into ∼600 bp fragments. Sonicated chromatin was immunoprecipitated for 2 h at 4°C, by 1.5 μg of an α-HA rat antibody (clone 3F10, Roche), 2.5 μg of an α-RNApolII mouse antibody (clone 4H8, Abcam), or 2.5 μg of murine IgG (Invitrogen), in a final volume of 100 μl. Immunoprecipitated DNA was purified according to the manufacturer's instructions. Lastly, 1/30 of each immunoprecipitated (IP) DNA sample (case α-HA-IP) or 1/60 of it (case α-RNApolI-IP and IgG-IP controls) were amplified by qPCR. For each sample, qPCRs were performed in technical triplicate. Averages were normalized against input chromatin and further normalized against NMHV/MF6-øor NMHV/MF2-øcontrols, in case of *NAP22* and *Emx2* tests, respectively. Experiments were performed at least in biological triplicate and analyzed by Student's t-test. Oligos were as in Supplementary Table S4.

### Immunofluorescence

HEK293T cells, naive or lipofected with LV_NMHV or LV_NMHV/pcDNA3ΔPvuII-MF6-NAP22, were grown on poly-L-lysine coated glass coverslips in 12 multiwell plates, fixed with 4% paraformaldehyde (PFA) for 20 min at 4°C and then washed three times in 1X PBS. Lentivirus-transduced, floating neural precursor aggregates were gently trypsinized to single cells and left to attach 1 h at 37°C to poly-L-lysine (200 μg/μl) coated SuperFrost Plus microscope slides (Menzel-Glaser). Here they were fixed by 4% PFA for 20 min at 4°C and washed three times in 1X PBS.

In all cases, immunofluorescence was performed as previously described (6). The following primary antibodies were used: anti-HA, rat clone 3F10 (Roche #12158167001), 1:500; anti-beta-actin, mouse clone AC-15 (Sigma #A3854, 1:1000); anti H3K9me3, rabbit polyclonal (Invitrogen #P7N49–1008), 1:200; anti-Tubb3, mouse clone Tuj1 (Covance #MMS-435P), 1:1000. Secondary antibodies were conjugates of Alexa Fluor 488, and Alexa Fluor 594 (Invitrogen), used at 1:600.

HA, beta-actin and H3K9me3 immunofluorescences were photographed on a Nikon Eclipse TI microscope, equipped with a 40X objective and a Nikon C2 confocal system. For each sample, Z-stacks of 6, 0.5 μm-spaced optical sections, flattened and averaged, were shown. Tubb3 immunofluorescences were photographed on a Nikon Eclipse TS100 fluorescence microscope equipped with a DS-2MBWC digital microscope camera with a 20X objective. For each independent biological replicate, at least six fields, corresponding to at least 1500 cells, were analyzed by an operator blind of cells ‘genotype’. Images were processed by Adobe Photoshop CS2 software^TM^. DAPI stained nuclei images were counted with ImageJ Cell Counter plug-in.

### Western blotting

Western analysis was performed according to standard methods. Total cell lysates in CHAPS buffer were quantified by BCA protein assay kit (Fisher Scientific # 10678484) and denatured at 95°C for 5 min, prior to loading. Fifty microgram of proteins was loaded per each lane and was run on a 12% acrylamide −0.1% SDS gel. NAP22 was detected by a primary rabbit anti-BASP1 polyclonal antibody (Sigma #SAB2107478), used at 1:2500, and a secondary HRP-conjugated anti-rabbit antibody (LifeTech # 32260), used at 1:2000. As previously reported (43), under these conditions NAP22 gives rise to a 50kDa dimer band. βACT was detected by a peroxydase C-conjugated mouse monoclonal antibody (Sigma # A3854), used 1:10 000. NAP22 and βACT were sequentially revealed by an ECL kit (GE Healthcare # GERPN2109). Images were acquired by an Alliance LD2–77.WL apparatus (Uvitec, Cambridge) and analyzed by Adobe Photoshop CS2 software^TM^ and Microsoft Excel 11 software^TM^.

### Statistical analysis

Each ‘biological replicate’ included cells pooled from at least two independent wells/petri dishes. Numbers of biological replicates analyzed in each experiment are shown under the corresponding graphs. Each biological replicate was scored at least in technical triplicate. Data were normalized as reported in figure legends, averaged and statistically evaluated by Student's t-test (unpaired, one-tail). Variability was graphically shown by standard error of mean bars.

## RESULTS

### Upregulation of endogenous genes by artificial, non-CRISPR, RNA-programmable transactivators

**Figure 1.**
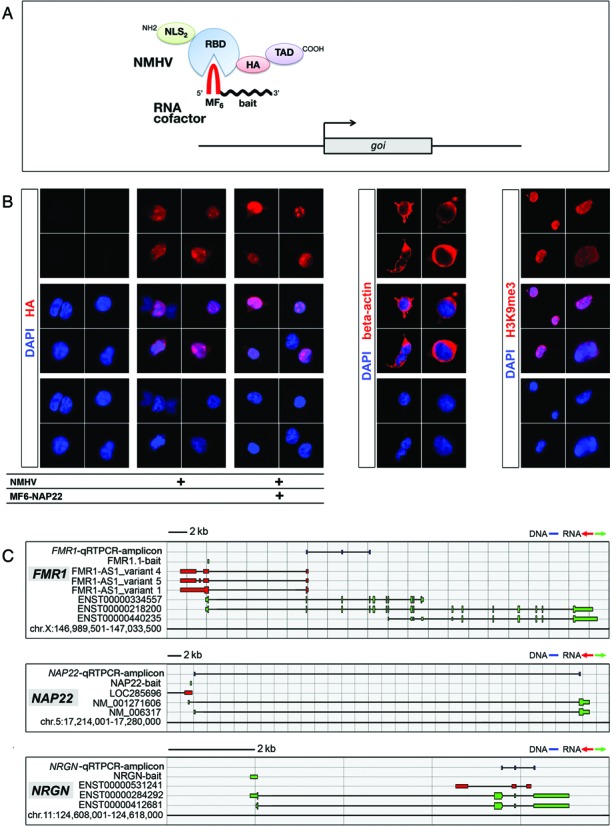

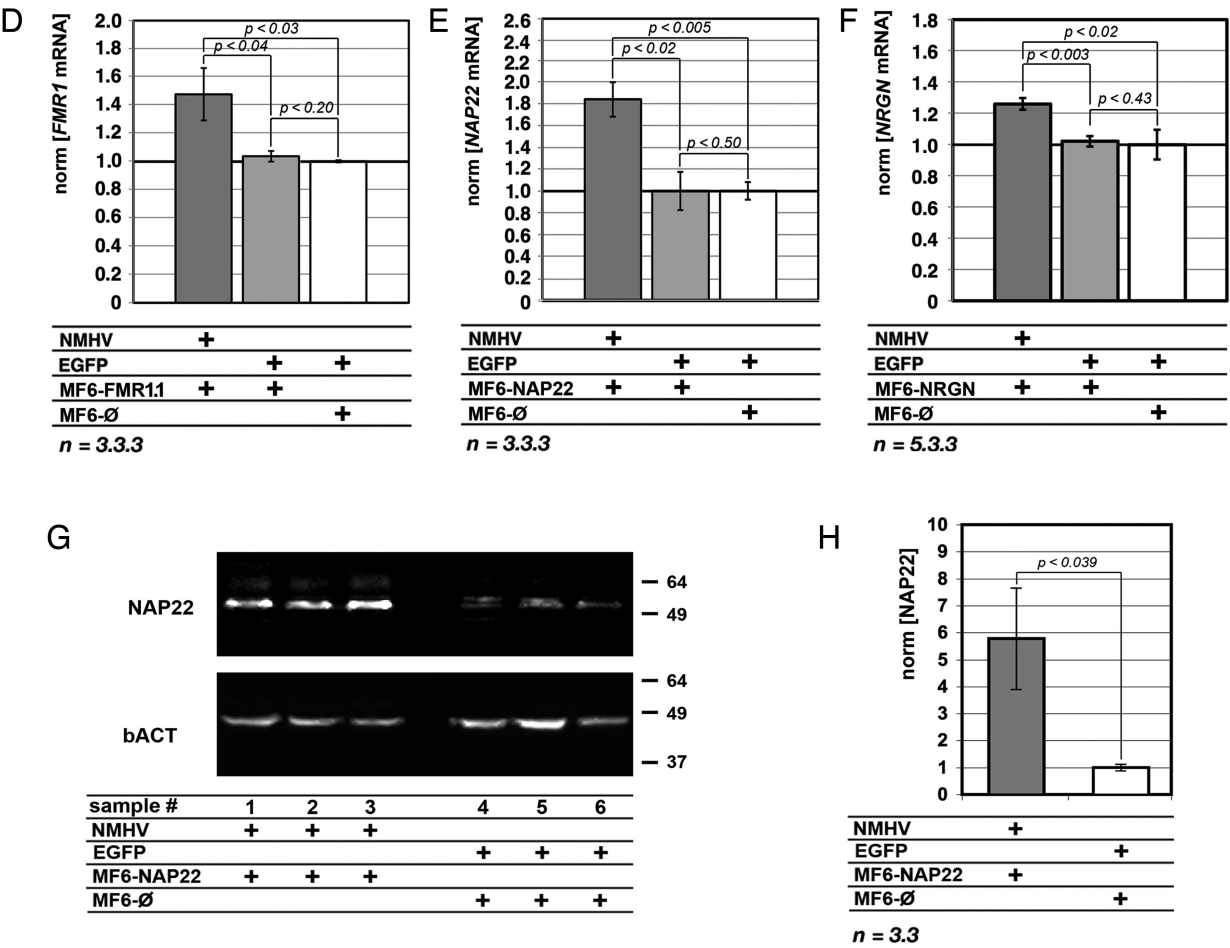
Design of the ribo-transactivator and its proof-of-principle validation. **(A)** Structure of the NMHV apo-activator and its RNA cofactor. NMHV includes: NLS2, nuclear localization signal 2x; RBD, MS2 RNA-binding domain; HA, hemagglutinin epitope and TAD, VP16-related transactivator domain, 3x. The RNA cofactor includes: MF6, MS2-high affinity, stem-and-loop finger, 6x; and ‘bait’, short, target gene specific, RNA tag. *GOI* is the GOI. **(B)** Subcellular distribution of NMHV in HEK293T cells, in the presence or in the absence of its MF6-NAP22 RNA cofactor, as revealed by anti-HA immunofluorescence. Beta-actin and H3K9me3 distributions, cytoplasmic and nuclear, respectively, are also shown, as references. **(C)** Schematics of human *FMR1*, *NAP22* and *NRGN* loci, with natural transcripts, artificial baits and diagnostic amplicons used in this study. Nucleotide numbering refers to UCSC-hg19. Color code: blu, DNA; green, sense-oriented RNA; red, antisense-oriented RNA. **(D–F)** Upregulation of *FMR1, NAP22* and *NRGN* mRNAs in HEK293T cells cotransfected with NMHV- and MF6-bait-encoding plasmids, as evaluated 72 h post-transfection. Pgkp1-EGFP (‘EGFP’) and MF6-øplasmids were used as controls. Results were normalized against *GAPDH* and further normalized against the EGFP/MF6-øcombination. **(G, H)** Upregulation of NAP22 protein by NMHV/MF6-NAP22. (**G**) Western blotting of NAP22 in HEK293T cells, four days after transfection by NMHV/MF6-NAP22 or EGFP/MF6-ø, via the ‘Lipofectamine 3000 protocol’. (**H**) Quantification of NAP22 protein detectable in (G). Results were normalized against βACT and further normalized against the EGFP/MF6-øcombination. Numbers of biological replicates, *n*, are displayed under the graphs. Bars represent s.e.m.'s.

To overactivate our GOIs by a programmable device, we first assembled a chimeric cDNA encoding for a novel polypeptidic apo-transactivator, NMHV (Figure 1A). This included an RNA binding domain (RBD) as well as a transactivating domain (TAD). The former corresponds to the V75E;A81G mutant version of the bacteriophage MS2 coat protein, with a low tendency to aggregate as well as an ability to bind its mRNA with high affinity (25). The latter consisted of three tandemly arranged F-type domains from the VP16 protein of the herpes simplex virus (44). To target the apo-transactivator to the cell nucleus, RBD was preceded by two copies of the SV40-T protein nuclear localization signal (NLS) (45). To allow immunodetection of the resulting polypeptide, a monomeric A influenza virus hemagglutinin tag (HA) (46) was interposed between RBD and TAD.

Then, for each GOI we built the DNA copy of a dedicated non-coding RNA cofactor (Figure 1A). This included a hexameric, MS2 coat protein-binding stem-and-loop moiety (26,27) (MF6) and a mid-sized (around 120 bases long), gene-specific RNA bait, complementary to a region near the GOIs TSS and co-oriented with its mRNA.

As a proof of principle, we decided to test our device in HEK293T cells, on three genes, *FMR1*, *NAP22* and *NRGN*, robustly expressed by these cells (http://webserver.mbi.ufl.edu/∼shaw/293.html). We built the cDNAs encoding for the NMHV apo-transactivator and the MF6-containing cofactors specific for these genes (Figure 1C and Supplementary Table S1). We cloned them into RNA polymerase II (RNApolII) expression vectors, downstream of the constitutive *Pgk1* and CMV promoters, respectively. Next, we assessed the capability of the NMHV polypeptide to enter cell nuclei (Figure 1B) and we set up a DNA delivery protocol, suitable to cotransfect a large fraction of HEK293T cells in the presence of limited signs of toxicity (Supplementary Figure S1).

Later, we co-delivered NMHV, MF6-FMR1, MF6-NAP22 and MF6-NRGN plasmids (as well as their controls) to HEK293T cells, in different combinations. After three days, the transfected cells were profiled for the three GOIs by qRTPCR (Figure 1C–F). Each NMHV/ncRNA cofactor pair consistently stimulated the corresponding GOI. Upregulation was 47±18% for *FMR1* (*P* < 0.030), 83±16% for *NAP22* (*P* < 0.004) and 25±4% for *NRGN* (*P* < 0.014) (Figure 1D–F). Conversely, overexpression of the three ncRNA cofactors in the absence of NMHV did not elicit any significant effect (Figure 1D–F) and the replacement of the VP16 domain by an EGFP moiety abolished gene upregulation (Supplementary Figure S2). Interestingly, NMHV and MF6-NAP22 delivery to HEK293T cells also led to a robust upregulation of NAP22 protein (5.77±2.03-fold, *P* < 0.039, *n* = 3.3; see Figure 1G and H).

Remarkably, gene transactivation was specific. In fact, each NMHV/ncRNA pair, while stimulating the corresponding GOI, did not affect the other two genes (Supplementary Figure S3). Moreover, selected genes sharing extensive homologies with the three GOIs in the surroundings of their TSSs (Supplementary Table S5) were not affected (Supplementary Figure S3). Transactivation did not spread along the chromosome far from the intended bait target (Supplementary Figure S4a). Conversely, it was restricted to transcription units having their TSSs located in the 5′ and 3′ surroundings of such target (Figure 1C–E and Supplementary Figure S4b). Interestingly, the outcome of the manipulation depended on bait orientation. While sense-oriented RNA baits consistently supported NMHV-dependent GOI transactivation (Figure 1C–F and Supplementary Figure S4b), the antisense-oriented ones often failed to achieve this effect (Supplementary Figure S4c).

**Figure 2.**
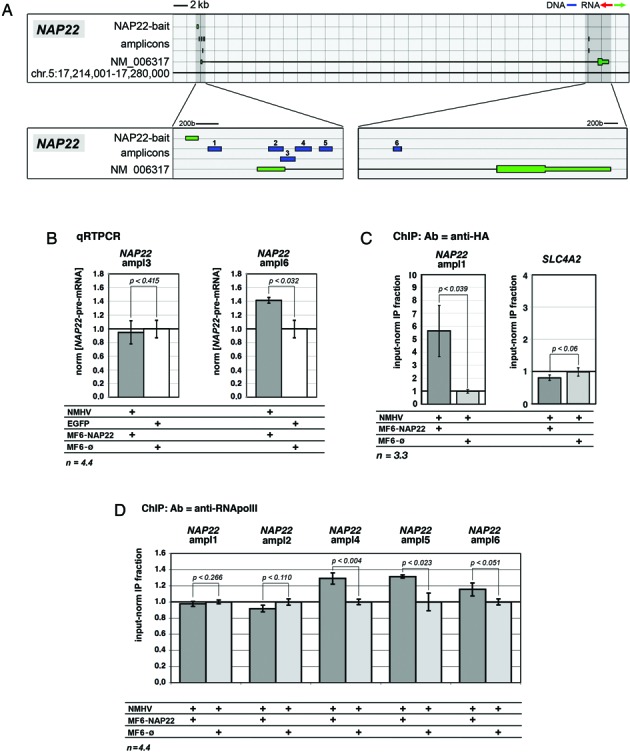
Mechanisms underlying *NAP22* transactivation in HEK293T cells. (**A**) Schematics of the *NAP22* locus with NAP22-bait and diagnostic amplicons employed for the analysis. (**B**) Modulation of *NAP22*-pre-mRNA in NMHV/MF6-NAP22-expressing cells, evaluated by intronic qRTPCR. Results were normalized against *GAPDH* and further normalized against the EGFP/MF6-øcombination. **(C)** Recruitment of NMHV at *NAP22* promoter in NMHV/MF6-NAP22-expressing cells, evaluated by anti-HA-ChIP/qPCR. The potential *NAP22*-offtarget *SLC4A2* was used as a specificity control. (**D**) RNApolII binding at different sites of the *NAP22* locus in NMHV/MF6-NAP22-expressing cells, evaluated by anti-RNApolII-ChIP/qPCR. In (C, D) results were normalized against input chromatin and further normalized against the NMHV/MF6-øcontrol. Noticeably, in (B, D) cells were transfected by the ‘Lipofectamine 3000 protocol’. In (B-D) cell culture timing was as described in Figure 1. Numbers of biological replicates, *n*, are displayed under the graphs. Bars represent s.e.m.'s.

To corroborate these findings and explore mechanisms mediating transactivation, we monitored the level of *NAP22*-pre-mRNA in HEK293T cells cotransfected with NMHV and, alternatively, MF6-NAP22 or its negative control. [Here, to ease the detection of consequences of our manipulations, we employed a further optimized transfection protocol (Supplementary Figure S5)]. We found that, upon delivery of NMHV and MF6-NAP22, the qRTPCR signal corresponding to distinct regions of *NAP22*-pre-mRNA was differentially affected. This signal was unchanged across the splice donor site, while it was upregulated by +41±4% (*P* < 0.032, *n* = 4.4) closer to the splice acceptor site (Figure 2A and B).

Moreover, we evaluated the enrichment of *NAP22* chromatin for select transcription effectors. We employed two antibodies, recognizing the HA epitope and the RNApoIII carboxyterminal domain. As for anti-HA ChIP, MF6-NAP22 expression enriched the region immediately downstream of the bait target (Figure 2A) by 5.5±2.0-fold, compared to MF6-ø(*P* < 0.039, *n* = 3.3) (Figure 2C). With the same antibody, no enrichment was observed for chromatin of *SLC4A2* (Figure 2C), a potential off-target of MF6-NAP22 (Supplementary Table S5). Concerning anti-RNApolII-ChIP, no change of the IP fraction was detected in the region between the NAP22-bait target and the *NAP22* splice donor. However, a moderate statistically significant enrichment was observed at three more distal sites, by the 5′ and 3′ ends of the intron (+29±7%, *P* < 0.005, *n* = 4.4; +32±2%, *P* < 0.023, *n* = 3.3 and +16±8%, *P* < 0.051, *n* = 3.4; respectively; see Figure 2A, D and Supplementary Figure S6).

All this suggests that, in the presence of the MF6-NAP22 cofactor, NMHV specifically binds to the surroundings of *NAP22*-TSS. In turn, this seems to promote the progression of RNApolII from the region immediately downstream of the TSS toward the 3′ end of the gene and results in increased transcription.

### Specific upregulation of brain patterning genes in pallial precursors by dedicated RNA-programmable transactivators and its biological consequences

**Figure 3.**
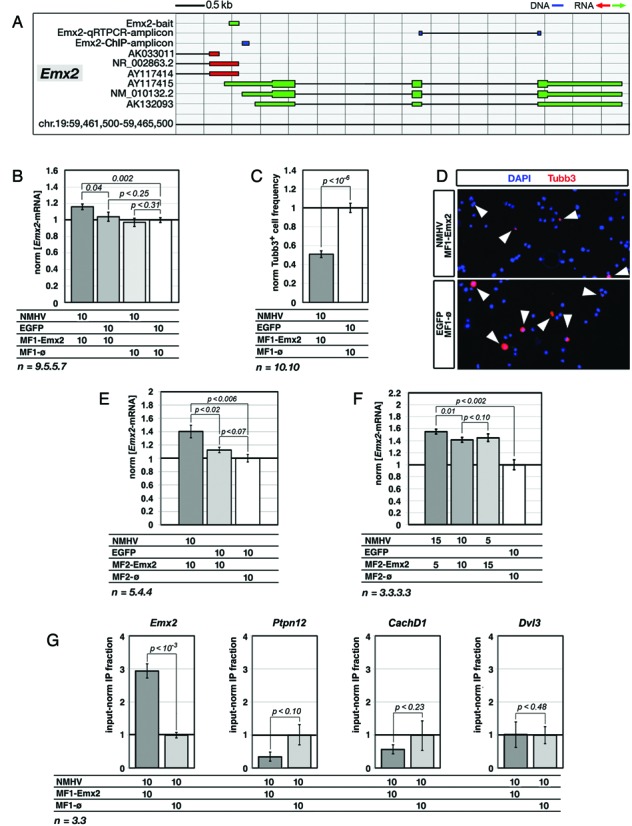
NMHV-mediated transactivation of *Emx2* in murine, embryonic cortico-cerebral precursors and its molecular and biological correlates. **(A)** Schematics of the murine *Emx2* locus, with natural transcripts, artificial baits and diagnostic amplicons used in this study. Nucleotide numbering refers to UCSC-mm10. Color code: blu, DNA; green, sense-oriented RNA; red, antisense-oriented RNA. **(B)***Emx2*-mRNA upregulation in precursors infected by NMHV- and MF1-Emx2-encoding lentivectors. EGFP and MF1-øviruses were used as controls. Results were normalized against *Gapdh* and further normalized against the EGFP/MF1-øcombination. **(C)** Reduced frequency of cells immunopositive for the neuron-specific Tubb3 marker, in cultures of NMHV/MF1-Emx2-overexpressing precursors. Results were normalized against EGFP/MF1-øcontrols. **(D)** Examples of aTubb3 immunofluorescences referred to in (C). **(E)** Enhancing *Emx2* transactivation by improving the RNA cofactor structure. The assay was run similar to Figure 2A, replacing the monomeric-finger ‘MF1-bait’ cofactor by its dimeric-finger ‘MF2-bait’ derivative. **(F)** Enhancing *Emx2* transactivation by increasing the m.o.i. ratio of NMHV- and MF2-Emx2-encoding lentiviruses. The best results were obtained by an NMHV/MF2-Emx2 ratio of 3:1. **(G)***Emx2* promoter enrichment in chromatin of NMHV/MF1-Emx2-overexpressing precursors, immunoprecipitated by an anti-HA antibody. Results were normalized against input chromatin and further normalized against the NMHV/MF1-øcontrol. Promoters of potential *Emx2*-offtargets *Ptpn12, CachD1 and Dvl3* were used as specificity controls. In all cases (B-G), cortico-cerebral cells were dissociated from E12.5 embryos, acutely infected, cultured for 96 h and finally analyzed. Throughout the figure, lentiviral multiplicities of infection (moi's) and numbers of biological replicates, *n*, are displayed under the graphs. Bars represent s.e.m.'s.

Encouraged by these results, we decided to test the portability of this strategy to a gene mastering cortico-cerebral histogenesis, in an established *in vitro* model of this process. We stimulated the embryonic patterning gene *Emx2* in high-density, primary cultures of murine, cortico-cerebral precursors. Due to poor transfectability of these cells, we moved to lentiviral vectors. As the hexameric MF6 moiety resulted unstable in our lentiviral backbone (42), we replaced it by its monomeric MF1 version. To MF1 we added an *Emx2*-specific bait, obtaining the MF1-Emx2 cofactor. We co-delivered lentiviruses encoding for NMHV, MF1-Emx2 and their controls to *Emx2*-expressing (47), murine embryonic day 12.5 (E12.5) pallial precursors, in different combinations. Each virus was administered at a multiplicity of infection (moi) of 10, which is sufficient to transduce the almost totality of neural precursors, regardless of their rostro-caudal identity (see Supplementary Figure S4 of (34) and Supplementary Figure S7 of the present paper). Four days later, infected cells were profiled for *Emx2*-mRNA by qRTPCR (Figure 3A). Compared to controls, coexpression of NMHV and MF1-Emx2 upregulated *Emx2* by 16% (*P* < 0.002, *n* = 9.7). Neither NMHV nor MF1-Emx2 alone elicited any significant effect (Figure 3B). Moreover, compared to uninfected cells, co-infection of precursors with both control viruses was ineffective (+2.4%, with *P* < 0.36 and *n* = 5.6, Supplementary Figure S8).

It has been reported that upregulation of *Emx2* in pallial precursors slows down their neuronal differentiation (6,30,33). To assess biological relevance of the small *Emx2* upregulation elicited by NMHV and MF1-Emx2, we evaluated the frequencies of cells expressing Tubb3 (an early neuronal postmitotic marker) within engineered neural cultures (Figure 3C, D and Supplementary Figure S9). Compared to controls, NMHV and MF1-Emx2 halved the frequency of Tubb3^+^ cells (*P* < 10^−6^, with *n* = 10.10), meaning that gene upregulation obtained by our strategy, albeit small, can yield robust biological effects.

We suspected that the limited *Emx2* upregulation elicited could be due to suboptimal interaction between the apo-transactivator and its ncRNA cofactor. Therefore, we tried to improve performances of our device, addressing this issue in two different ways. First, it has been reported that RNAs containing increasing numbers of MS2-binding stem-and-loop fingers interact with the MS2 coat protein in a progressively stronger way (48). Therefore, we replaced the monomeric-finger MF1-Emx2 cofactor by its dimeric-finger MF2-Emx2 derivative and assessed the performance of such derivative, paired with NMHV. Interestingly, this device increased *Emx2*-mRNA by about 40% (*P* < 0.005, *n* = 5.4), while MF2-Emx2 alone did not elicit any significant effect (Figure 3E). Second, we hypothesized that non-optimal apo-transactivator/RNA cofactor ratios might jeopardize the outcome of the system, because of defective holo-transactivator formation and possible dominant-negative effects. So we modulated the LV_NMHV/LV_MF2-Emx2 moi's ratio, while keeping the total moi fixed. When this ratio equaled 3:1, *Emx2*-mRNA was increased by 55% (*P* < 0.002, *n* = 3.3) (Figure 3F).

To assess if MF2-Emx2/NMHV-dependent stimulation was restricted to *Emx2*, we monitored the expression levels of six other genes (*Pax6*, *Hes6*, *Sip1*, *Couptf1*, *Nf1a* and *Lhx2*), randomly chosen among those active in pallial precursors (49), that were potentially accessible to exogenous transactive complexes. None were affected, when neural cells were challenged by the best *Emx2*-transactivating strategy described above (Supplementary Figure S10a). Four additional genes (*Arid1a, CachD1, Ptpn12* and *Dvl3*), sharing extensive homologies with *Emx2* in the surroundings of their TSSs (Supplementary Table S6), were neither affected (Supplementary Figure S10a). Notably, the replacement of the original ncRNA bait with its reverse complementary counterpart abolished *Emx2* transactivation (Supplementary Figure S11a).

To corroborate these findings, we performed a ChIP analysis. We immunoprecipitated chromatin of neural precursors infected with LV_NMHV and, alternatively, LV_MF1-Emx2 or its negative control, by an anti-HA antibody. Then, we quantified the genomic region downstream of the *Emx2*-bait by qPCR (Figure 3A). LV_MF1-Emx2 expression enriched this region by 3.0±0.2, compared to LV_MF1-ø(*P* < 0.0005, *n* = 3.3). No enrichment was conversely observed for the chromatin of three potential LV_MF1-Emx2 off-targets, *Ptpn12*, *CachD1* and *Dvl3* (Figure 3G and Supplementary Table S6). This suggests that NMHV physically and specifically interacts with the *Emx2* locus in living cells, depending on the *Emx2*-bait, and possibly stimulates *Emx2* transcription.

**Figure 4.**
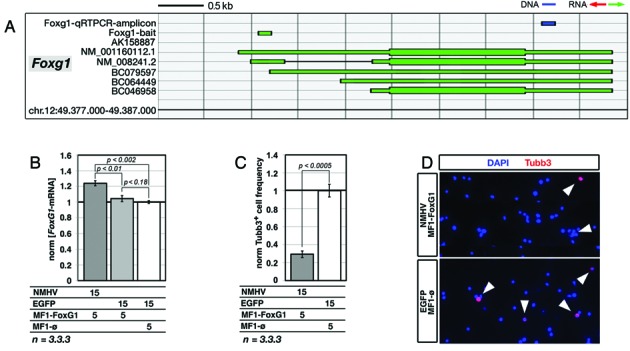
NMHV-mediated transactivation of *Foxg1* in murine, embryonic cortico-cerebral precursors and its biological correlate. **(A)** Schematics of the murine *Foxg1* locus, with natural transcripts, artificial baits and diagnostic amplicons used in this study. Nucleotide numbering refers to UCSC-mm10. Color code: blu, DNA; green, sense-oriented RNA. **(B)***Foxg1*-mRNA upregulation in precursors infected by NMHV- and MF1-Foxg1-encoding lentivectors. EGFP and MF1-øviruses were used as controls. Results were normalized against *Gapdh* and further normalized against the EGFP/MF1-øcombination. **(C)** Reduced frequency of cells immunopositive for the neuron-specific Tubb3 marker, in cultures of NMHV/MF1-Foxg1-overexpressing precursors. **(D)** Examples of aTubb3 immuno-fluorescences referred to in (C). In all cases (B–D) time-frame of the experiments as well as representation of moi's and statistical parameters are as in Figure 3.

To generalize these results, we built a ncRNA cofactor specific for another key gene involved in brain patterning, *Foxg1* (Figure 4A). Coexpression of NMHV and MF1-Foxg1 cofactor in murine pallial precursors upregulated *Foxg1* by 23% (*P* < 0.002, *n* = 3.3) (Figure 4B). This led to a pronounced decrease of newborn, Tubb3^+^ neurons generated by the engineered culture (about −70%, with *P* < 0.0004 and *n* = 3.3) (Figure 4C and D). Transactivation was restricted to the intended transcription unit. Neither the structurally unrelated *Emx2* nor the *Foxg1*-cis-associated AK158887 and 3110039M20Rik-001 units were affected (Supplementary Figures S10b and S11b).

**Figure 5.**
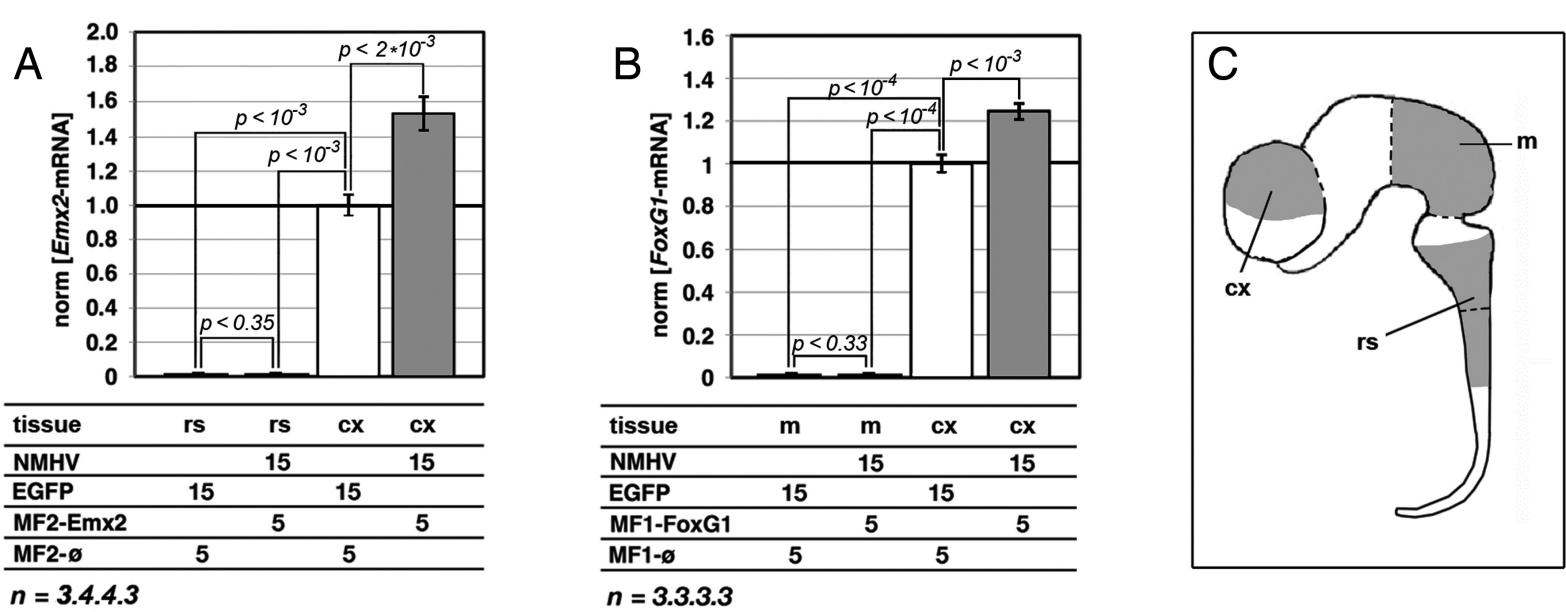
Lack of responsivity to *Emx2-* and *FoxG1-*specific ribo-transactivators by neural precursors of non-cortico-cerebral origin. **(A)** Unchanged *Emx2*-mRNA levels in E10.5 rhombo-spinal precursors, infected by NMHV- and MF2-Emx2-encoding lentiviruses or EGFP- and MF2-øcontrols. **(B)** Unchanged *FoxG1*-mRNA levels in E10.5 mesencephalic precursors, infected by NMHV- and MF1-FoxG1-encoding lentiviruses or EGFP and MF1-øcontrols. In both (A) and (B), transactivation of the two genes in E12.5 cortico-cerebral precursors is shown, as a positive control. Moreover, in both cases, results were normalized against *Gapdh* and further normalized against E12.5 cortico-cerebral precursors treated by EGFP/MF2-øand EGFP/MF1-øcombinations, respectively. Time frame of the experiments as well as representation of moi's and statistical parameters is as in Figure 3. **(C)** Idealized representation of the murine E10.5 neural tube. cx, cerebral cortex; m, mesencephalon; rs, rhombo-spinal tract.

Lastly, to better define the prospective scope of application of our device, we considered its effectiveness in correlation to the baseline expression level of the GOI. The question arose since our ncRNA cofactors did not harbor long polypurine/polypyrimidine tracts required for RNA:DNA triple helix formation (50). For this reason, the interaction of these cofactors with chromatin could require a transcriptionally active conformation, prone to alternative mechanisms of ncRNA tethering (such as ncRNA:DNA heteroduplexing or ncRNA docking to nascent RNAs). To preliminarily address this issue, we delivered our best performing *Emx2*- and *Foxg1*-promoting protocols to neural precursors taken from two regions of the embryonic neural tube which do not express these two genes, rhombo-spinal tract (47) and mesencephalon (51), respectively. As expected, GOI-mRNA levels remained extremely low as compared to cortico-cerebral precursors. No gene upregulation was elicited at all (Figure 5A–C).

### Shortening the baits and assessing mismatch tolerance of our device

**Figure 6.**
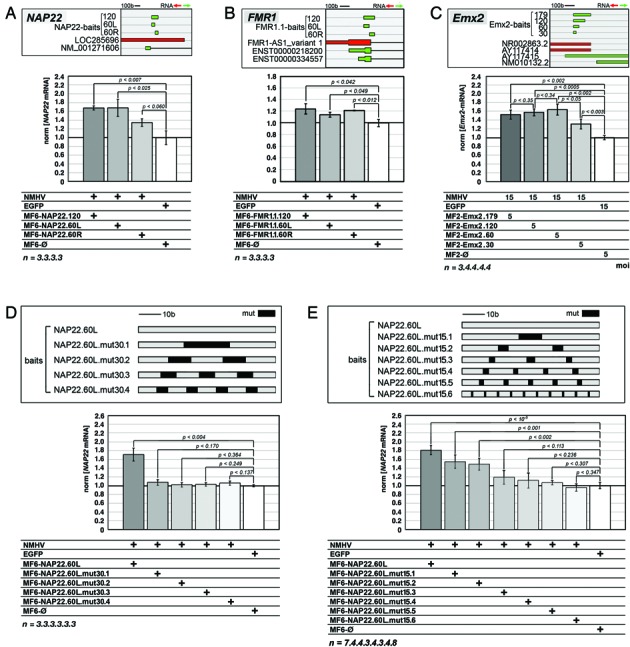
Consequences of RNA-bait shortening and mutagenesis. **(A-C)** Comparison of NMHV-dependent gene activation driven by select ‘primary’ baits, NAP22 (A), FMR1.1 (B) and Emx2-S (C), and ‘secondary’ baits obtained by heminested shortening of the former ones. **(D, E)** Full or partial suppression of NMHV-dependent *NAP22* transactivation driven by the NAP22.60L bait, upon replacement of 30% (D) or 15% (E) of its original bases by mutant ones. Mutant bases were distributed in up to four (D) and up to 10 (E) equispaced mismatching modules, as shown in top panels (sequences of mutant baits are reported in Supplementary Table S7). Results were normalized against *GAPDH* (A, B, D, E) and *Gapdh* (C) and further normalized against EGFP/MF6-ø(A, B, D, E) and EGFP/MF2-ø(C) control samples. Time frame of the experiments as well as representation of moi's and statistical parameters was as in Figures 1 (A, B, D, E) and 3 (C).

To further optimize our device, we shortened the RNA bait in charge of recognizing the GOI. This would minimize off-target risks and it would make chemical synthesis of baits for therapeutic applications easier. We replaced the NAP22 and FMR1.1 baits, sense-oriented and approximately 120b long, with their respective 5′ and 3′ halves (.60L and .60R, respectively, see Supplementary Table S1), and assayed the corresponding MF6-containing cofactors with the NMHV apofactor, in HEK293T cells. All four baits were able to support transactivation of the intended target genes (Figure 6A and B).

To confirm this result, additional tests were run on the *Emx2*-activating device, in cortico-cerebral precursors. Here, reducing the ncRNA bait from 179 to 60 bases (Supplementary Table S1) did not jeopardize device activity, which was slightly increased (Figure 6C). Further halving of bait length reduced such activity to hemi-maximal values (Figure 6C), suggesting that 60 base-long baits could be a satisfactory tool for gene stimulation.

Finally, to further confirm the specificity of our device, we mutagenized the most effective ‘.60-type’ bait referred to above, NAP22.60L, and assessed the performances of the resulting MF6 chimaeras. Replacement of 30% of the original bases by mutant ones, distributed in one to four equispaced mismatch modules, fully suppressed *NAP22* transactivation (Figure 6D and Supplementary Table S7). Conversely, replacement of 15% of these bases resulted into a more articulated pattern. Transactivation was still observed when the baits included fully homologous modules of ≥18 bases. It was completely abolished as the length of these modules fell below 14 (Figure 6E and Supplementary Table S7).

## DISCUSSION

Here we describe a novel ribonucleoproteic transactivator able to stimulate expression of endogenous genes *ad libitum*. This device was able to enter the nucleus and interact with the GOI chromatin through its RNA cofactor. By means of it, we specifically upregulated five independent genes, in cell lines as well as in primary cultures of murine pallial precursors. Such upregulation was small. However, it was sufficient to trigger an appreciable biological effect. Remarkably, activity of this device was restricted to cells where the GOI is normally transcribed. Finally, it was possible to improve this transactivator, by optimizing the interaction between polypeptidic and RNA components of it as well as reducing the size of the GOI-specific RNA bait.

The expression gains we observed primarily in HEK293T cells were +47±18% for *FMR1*, +83±16% for *NAP22* and +25±4% for *NRGN*. We are aware that random fluctuations of cell cultures upon experimental manipulation and trivial off-target effects could contribute to them. However, a high bait-target homology was needed to get mRNA upregulation (Supplementary Figures S3, S10 and Figure 6). Moreover, results were consistently reproducible over a number of biological replicates (e.g.: MF6-NAP22.60L-dependent *NAP22* upregulation, shown in Figure 6A,D,E, and MF1-Emx2-dependent *Emx2* upregulation, in Figure 3B and Supplementary Figure S11a). All that suggests that these expression gains were largely due to specific gene stimulation.

Notably, the results referred to above were obtained upon cotransfection of about only one-third of the total HEK293T cell population (Figure 1 and Supplementary Figure S1). Likewise, the best upregulation elicited in neural precursors (+55±4%, for *Emx2*) was detected in a cell population infected at moi's of 15 and 5 (Figure 3F) and therefore cotransduced at only 75% (see Supplementary Figure S3 of (34)). When the transfected HEK293T cell fraction was doubled (thanks to an optimized protocol), *NAP22* and *NRGN* upregulation increased up to +143±17% and +38±12%, respectively (Supplementary Figure S5).

However, incomplete cell population transduction could not be the only cause of limited gene expression gain and additional issues likely contributed to it. Among them there is the poor stability of our apoenzyme/coenzyme complex. In fact, better performances were elicited, as the number of MF fingers included into the RNA cofactor increased (Figures 1D–F, 3B,E and 4B). In addition, substantial benefits could be achieved by appropriately modulating the apofactor/cofactor ratio (Figure 3F). Second, the limited power of our device could reflect suboptimal interaction between the transactivator and its target gene. In this respect, a stronger transactivation might be achieved by targeting the same gene with multiple baits. On the other hand, the possibility of implementing a multi-gene overexpression program by a polygenic bait should be explored.

Even if *Emx2* and *Foxg1* upregulation obtained in this study was <2-fold, it was sufficient to robustly change the behavior of neural precursors (Figures 3C,D and 4C,D). This occurred in dorsal telencephalic (i.e. pallial) precursors, kept as high-density floating cultures under growth factors. These conditions do not affect precursors’ positional identity (52,53), stimulate their mitotic activity and largely recreate the complexity of cell–cell interactions characterizing proliferative layers of the developing brain (34). The responsivity that neural cells showed to even subtle variations of gene expression levels was not surprising. In fact, similar findings were previously reported for *Emx2* and other genes involved in brain patterning (6,54,55). The inhibition of neuronal differentiation triggered by *Emx2* and *Foxg1* upregulation might be exploited to enlarge the proliferating neural pool, namely a result of obvious therapeutic interest (34).

Gene stimulation obtained by our device was specific, in at least three key aspects. First, it required the coexpression of both the NMHV apofactor and the GOI-cofactor. Removal of NMHV or replacement of the NMHV-VP16 moiety by an EGFP polypeptide fragment abolished it (Figures 1D–F, 3B,E,F, 4B and Supplementary Figure S2). [Actually, two baits out of eight working ones, FMR1.2 and NAP22-AS, upregulated the corresponding genes to some extent, even in the absence of the apofactor. However, in these two cases, gene upregulation was far more pronounced upon further NMHV expression (Supplementary Figure S4b and c). Moreover, NMHV reversed gene-downregulation induced by two antisense baits, MF6-NRGN-AS and MF1-Emx2-AS (Supplementary Figures S4c and S11a, respectively)].

Second, GOI stimulation required high homology between the RNA bait and its intended target sequence. Potential off-target genes, suitable to be transcribed and/or provided with partial homology to our GOIs in the surroundings of their TSSs, were not affected (Supplementary Figures S3 and S10 and Supplementary Tables S5 and S6). The RNA bait could be shortened up to 60 bases, in the absence of adverse effects (Figure 6A–C). Mutagenizing the 60-bp-long NAP22 bait by 30% fully abolished *NAP22* stimulation (Figure 6D and Supplementary Table S7). As the mutagenesis rate was lowered to 15%, transactivation was detectable only when the bait included fully matching modules longer than 17 bases (Figure 6E and Supplementary Table S7).

Third, the bait-dependent transactivating effect exerted by NMHV was tightly restricted to the surroundings of the bait target and depended on bait orientation. TSSs located >1 kb far from the bait target were not affected (Supplementary Figures S4a and S11b). Conversely, transcription units having their 5′ ends <0.5 kb far from the bait target displayed a more articulated behaviour. Upon NMHV overexpression, they were generally stimulated by sense-oriented baits (Figures 1C–G, 3A,B,E,F, 4A,B and Supplementary Figure S4b), while variably responding to antisense ones (Supplementary Figures S4c and S11a).

To summarize, as length, orientation and distance from TSS of the RNA bait were properly tuned, our device appeared to work specifically and reliably. As many as six primary baits out of six tested, 115–179 bases long, sense-oriented and directed against TSS-proximal targets (Figures 1C–F, 3A,B,E,F and 4A,B, and Supplementary Figure S4b), supported an appreciable and selective gene upregulation. Moreover, the same happened for six more baits, obtained from shortening of the primary ones (Figure 6A–C).

During the execution of this study, several groups reported the successful creation of a novel RNA-programmable transactivator type (18–23), originating from domestication of Type II, bacterial CRISPR adaptive immune system. Compared to CRISPR effectors, our NMHV apofactor shows three substantial differences. First, it is 7-fold smaller (24), which may facilitate its artificial synthesis and delivery. Second, it is considerably less powerful than CRISPR effectors (20). This may reflect the helicase activity intrinsic to CRISPR molecules (56), absent in ours. Third, CRISPR-DNA interactions are not prevented by repressive epigenetic marks, such as H3K27me3 (22) or 5meC (23,57), and CRISPR-stimulated genes include silent transcription units (21,23) (our data not shown). Conversely, our device only works with genes already expressed at sustained levels (Figure 5). These last two features obviously limit the general interest in our device. On the other side, they make it potentially suitable for specific applications, such as rescue of haploinsufficiencies.

In theory, the best performing RNA-baits used in this study, being co-oriented with the upregulated mRNAs (Figures 1C–F, 3A,B,E,F, 4A,B and Supplementary Figure S4b), might act as molecular decoys for possible antisense transcripts. However, except one case (Supplementary Figure S4b), gene upregulation elicited by our RNA cofactors generally required the presence of the bulky NMHV apofactor (Figures 1D–F, 3B,E,F, 4B and Supplementary Figure S2), which is at odds with the decoy hypothesis. Moreover, the majority of our working RNA cofactors, being co-oriented with their cognate mRNAs, cannot directly interact with them. This rules out any further possibility of post-transcriptional regulation. On the other side, ChIP data show that the NMHV polypeptide is recruited to chomatin in a RNA-cofactor-dependent way (Figures 2C and 3G). Moreover, the enrichment of 3′ regions of the GOI for RNApolII arises (Figure 2D). Finally, upregulation of mRNA is associated to increased levels of its pre-mRNA precursor (Figure 2B). Altogether, these data suggest that our device rather acts by promoting gene transcription.

We do not know how our transactivator recognizes its target and promotes transcription. Concerning gene recognition, should the RNA cofactor directly interact with its cognate DNA, this would hardly occur via a triple helix structure, as triple helix formation requires long homopurinic-homopyrimidinic traits, absent in our baits (50). Conversely, the RNA cofactor could bind to the DNA template—previously unwound by the transcription machinery—via Watson and Crick base pairing, leading to D-loop formation. Alternatively, gene recognition could be indirect, i.e. the RNA cofactor might be docked to chromosomes via nascent antisense RNA molecules, still tethered to the sense-TSS surroundings. Actually, antisense transcripts spanning sense-TSSs were documented at the *NAP22*, *FMR1* and *Emx2* loci, the most responsive to NMHV/RNA-cofactor stimulation (Figures 1C–F and 3A,B,E,F). Similar transcripts could have escaped detection at the *NRGN* and *Foxg1* loci, less responsive to NMHV/RNA-cofactor, because of their lower abundance (Figures 1C–F and 4A,B). Interestingly, docking of ncRNA co-transactivators to nascent antisense RNAs would nicely account for preferential requirement of sense-oriented RNA baits to achieve gene transactivation (Supplementary Figures S4c and S11a).

Regardless of the mechanism of chromatin recognition, RNA-mediated tethering of NMHV to 5′ TSS surroundings might promote transcription by a variety of VP16-dependent mechanisms (58–63). VP16 can promote both transcription initiation and pre-mRNA elongation (58). However, an antibody recognizing RNApolII enriched the immunoprecipitate from NMHV/MF6-NAP22-treated cells for *NAP22* intronic regions, but not for *NAP22*-TSS surroundings (Figure 2D). This is puzzling. It could reflect the fact that the gain in RNApolII progression driven by NMHV/MF6-NAP22 matched or exceeded the gain in RNApolII recruitment at the TSS, resulting in no net increase of RNApolII bound to the TSS region. No doubt, all these mechanistic aspects deserve further in-depth investigation.

In conclusion, here we described a novel programmable transactivator, including a polypeptidic effector and an RNA bait in charge of recognizing the intended target gene. We provided a basic characterization of its functional properties. We preliminarily addressed molecular mechanisms mediating its action. This device might be useful for some specific applications. Presently, however, it is a simple prototype, which still needs a substantial improvement of its transactivation power as well as a better characterization of its target specificity and mechanism of action. These issues will be hopefully subject of a dedicated follow-up study.

## Supplementary Material

SUPPLEMENTARY DATA
