# Supplementary material for: Upregulating endogenous genes by an RNA-programmable artificial transactivator
Source: Nucleic Acids Res. 2015 Jul 7;43(16):7850–64. doi: 10.1093/nar/gkv682 (PMC4652751; doi:10.1093/nar/gkv682)
Supplement: SUPPLEMENTARY DATA [file supp_43_16_7850__index.html]

Upregulating endogenous genes by an RNA-programmable artificial transactivator — Upregulating endogenous genes by an RNA-programmable artificial transactivator — SUPPLEMENTARY DATA 

# Upregulating endogenous genes by an RNA-programmable artificial transactivator

## SUPPLEMENTARY DATA

- SUPPLEMENTARY DATA
