# Supplementary material for: Upregulating endogenous genes by an RNA-programmable artificial transactivator
Source: Nucleic Acids Res. 2015 Jul 7;43(16):7850–64. doi: 10.1093/nar/gkv682 (PMC4652751; doi:10.1093/nar/gkv682)
Supplement: SUPPLEMENTARY DATA [file supp_gkv682_nar-00812-y-2014-File009.pdf]

SUPPLEMENTARY INFORMATION

[Upregulating endogenous genes by an RNA-programmable artificial transactivator, by Fimiani et al.]

Contents:

- SUPPLEMENTARY FIGURES S1-S11

- SUPPLEMENTARY TABLES S1-S7

SUPPLEMENTARY FIGURES

Supplementary Figure S1. Evaluation of HEK293T cell cotransfection efficiency. (a) Absolute frequencies of fluorescent elements, evaluated 72 hours after LipoD co-transfection of HEK293T cells with equimolar amounts of Pgkp1-mCherry (8.0kb) and CMVp-EGFP (4.7kb) plasmids. In *x*, total amounts of DNA delivered to 10^6 cells are indicated. **(b)** Surviving fractions of cells referred to in **(a)**. Data normalized against untransfected cells. **(c)** Dark and bright field pictures of cells referred to in **(a)**, transfected with 2 μg of total DNA /10^6 cells.

Figure S1

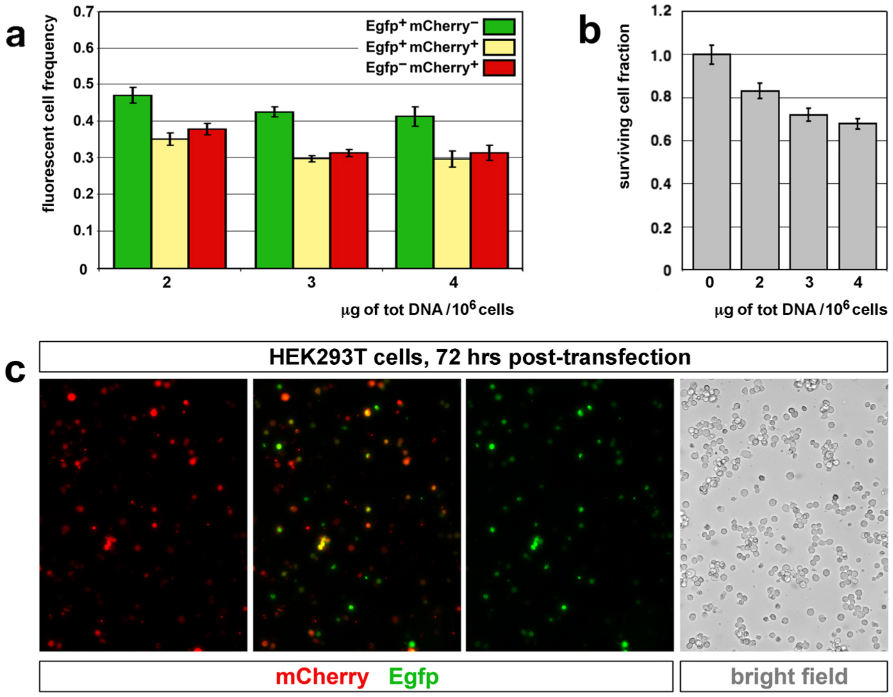

Supplementary Figure S2. Requirement of the VP16 domain for the NMHV function. *NAP22*-mRNA levels in HEK293T cells cotransfected by NMHV, NMHE, EGFP, MF6-NAP22 and MF6-Ø, in different combinations. Results were normalized against *GAPDH* and further normalized against EGFP/MF6-ø controls. Time-frame of the experiments and statistical parameters were as in Figure 1.

Figure S2

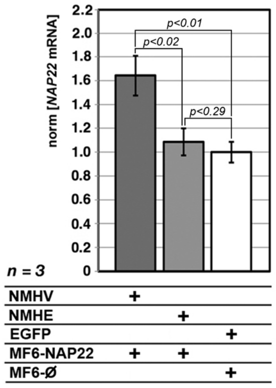

Supplementary Figure S3. Specificity of NMHV-mediated *FMR1, NAP22 and NRGN* transactivation: no perturbation of other active genes and structurally similar, potential offtargets. No aspecific gene upregulation in HEK293T cells expressing NMHV and the MF6-type, FMR1.1 **(a)**, NAP22 **(b)** or NRGN **(c)** baits. *FXR1*, *SLC4A2* and *EGR3* are potential offtargets, with 5' ends displaying remarkable homology to FMR1.1, NAP22 and NRGN baits, respectively (see Supplementary Table S5). Results were normalized against *GAPDH* and further normalized against EGFP/MF6-ø controls. Time-frame of the experiments and statistical parameters were as in Figure 1.

Figure S3

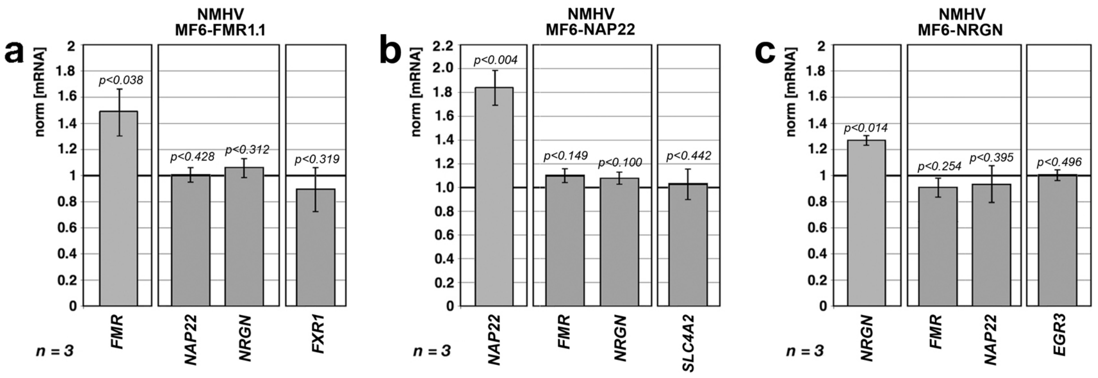

Supplementary Figure S4. Specificity of NMHV-mediated *FMR1*, *NAP22* and *NRGN* transactivation: limited cis-propagation and polarity effects. (a) Absent upregulation of transcripts stemming from TSSs >0.5kb far from bait-targets, in HEK293T cells co-expressing NMHV and the MF6-type, FMR1.1, NAP22 or NRGN baits. **(b)** Upregulation of transcripts originating from *FMR1*-TSSs, being <0.5kb far from bait-target, in HEK293T cells co-expressing NMHV and the *sense*-oriented, MF6-type, FMR1.2 bait. **(c)** Variable modulation of transcripts stemming from TSSs <0.5kb apart from bait-targets, in HEK293T cells co-expressing NMHV and the *antisense*-oriented, MF6-type, FMR1.1-AS, FMR1.2-AS, NRGN-AS or NAP22-AS baits. In each case, transcript upregulation by NMHV and the corresponding sense-oriented bait is shown as a reference. Time-frame of the experiments, data normalization and statistical parameters were as in Figure 1.

Figure S4

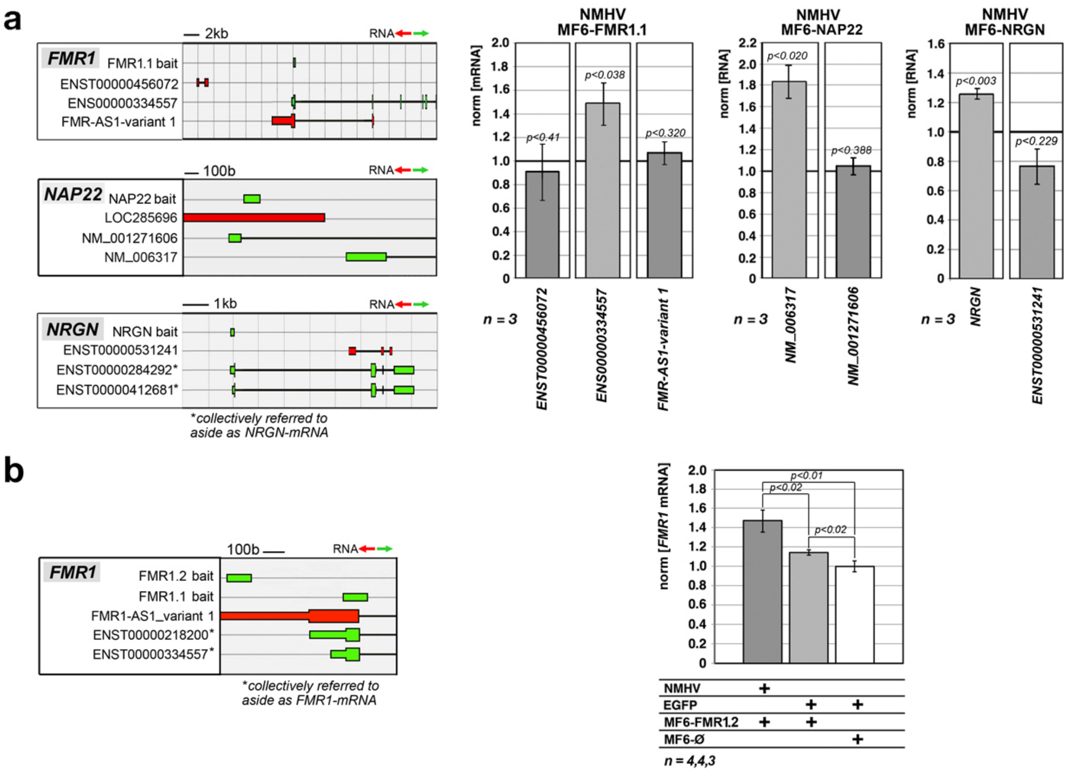

(split picture, to be continued)

Figure S4

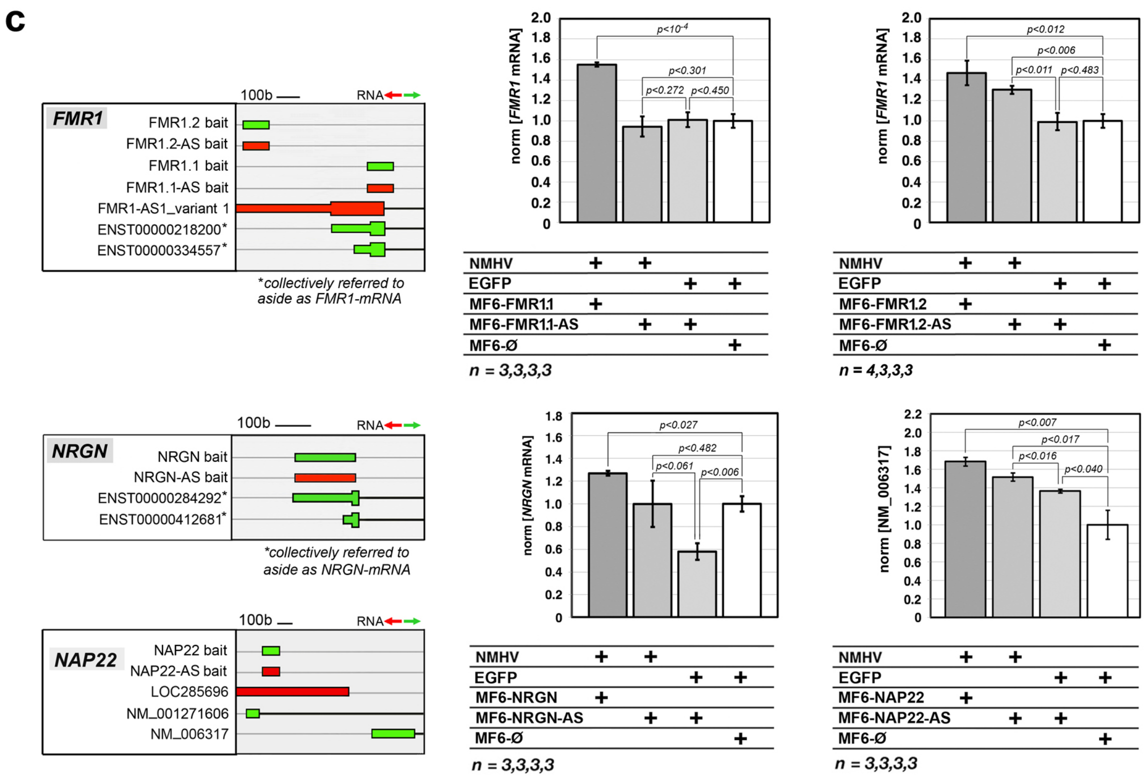
c
FMR1
FMR1.2 bait
FMR1.2-AS bait
FMR1.1 bait
FMR1.1-AS bait
FMR1-AS1_variant 1
ENST00000218200*
ENST00000334557*
100b
RNA
*collectively referred to aside as FMR1-mRNA
norm [FMR1 mRNA]
p<0.301
p<0.272
p<0.450
NMHV
EGFP
MF6-FMR1.1
MF6-FMR1.1-AS
MF6-Ø
n = 3,3,3,3
p<0.012
p<0.006
p<0.011
p<0.483
MF6-FMR1.2
MF6-FMR1.2-AS
n = 4,3,3,3
NRGN
NRGN bait
NRGN-AS bait
ENST00000284292*
ENST00000412681*
*collectively referred to aside as NRGN-mRNA
norm [NRGN mRNA]
p<0.027
p<0.482
p<0.061
p<0.006
MF6-NRGN
MF6-NRGN-AS
n = 3,3,3,3
NAP22
NAP22 bait
NAP22-AS bait
LOC285696
NM_001271606
NM_006317
norm [NM_006317]
p<0.007
p<0.017
p<0.016
p<0.040
MF6-NAP22
MF6-NAP22-AS
n = 3,3,3,3

Supplementary Figure S5 Improving the transfection efficiency of HEK293T cells results in a consistent increase of NMHV/MF6-bait-dependent gene transactivation. (a) Dark and bright field pictures of HEK293T cells co-transfected with equimolar amounts of Pgkp1-mCherry (8.0kb) and CMVp-EGFP (4.7kb) plasmids (2.5 μg of total DNA/10^6 cells). Analysis performed 72 hours after completion of the two-steps, Lipofectamine 3000 transfection procedure. **(b)** Absolute frequencies of fluorescent elements, referred to in **(a)**. **(c,d).** Upregulation of *NAP22* and *NRGN* mRNAs in HEK293T cells cotransfected with NMHV- and MF6-bait-encoding plasmids, according to the procedure shown in **(a)**. Pgkp1-EGFP ("EGFP") and MF6-ø plasmids were used as controls. Results were normalized against *GAPDH* and further normalized against the EGFP/MF6-ø combination. Numbers of biological replicates, *n*, are displayed under the graphs. Bars represent s.e.m.'s.

Figure S5

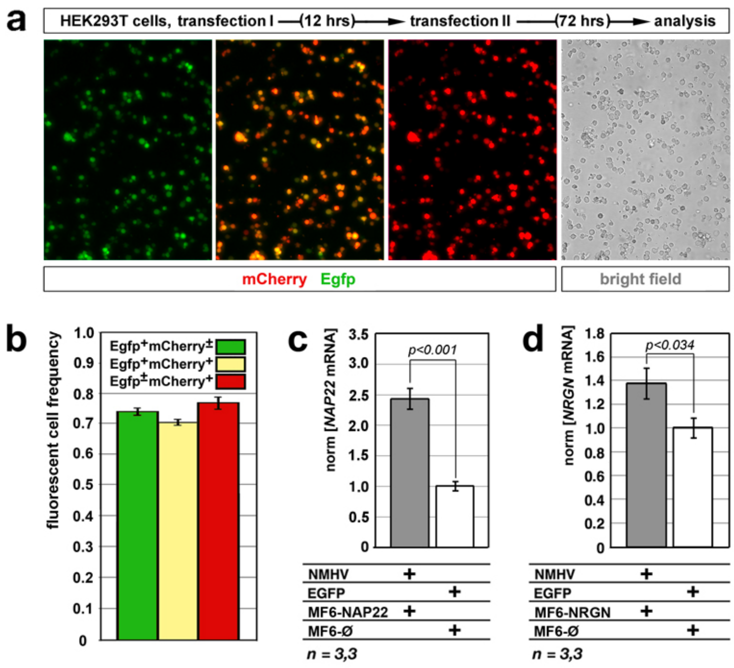

Supplementary Figure S6. Fraction of *NAP22* chromatin immunoprecipitated by anti-RNApolII, in HEK293T cells expressing NMHV/MF6-NAP22 or NMHV/MF6-ø. Graphs refer to results of the experiments described in Figure 2d, prior to normalization against NMHV/MF6-ø controls. They include results of additional control tests, run challenging NMHV/MF6-ø cells by IgGs, in place of anti-RNApolII. *n* are numbers of biological replicates. Bars represent s.e.m.'s.

Figure S6

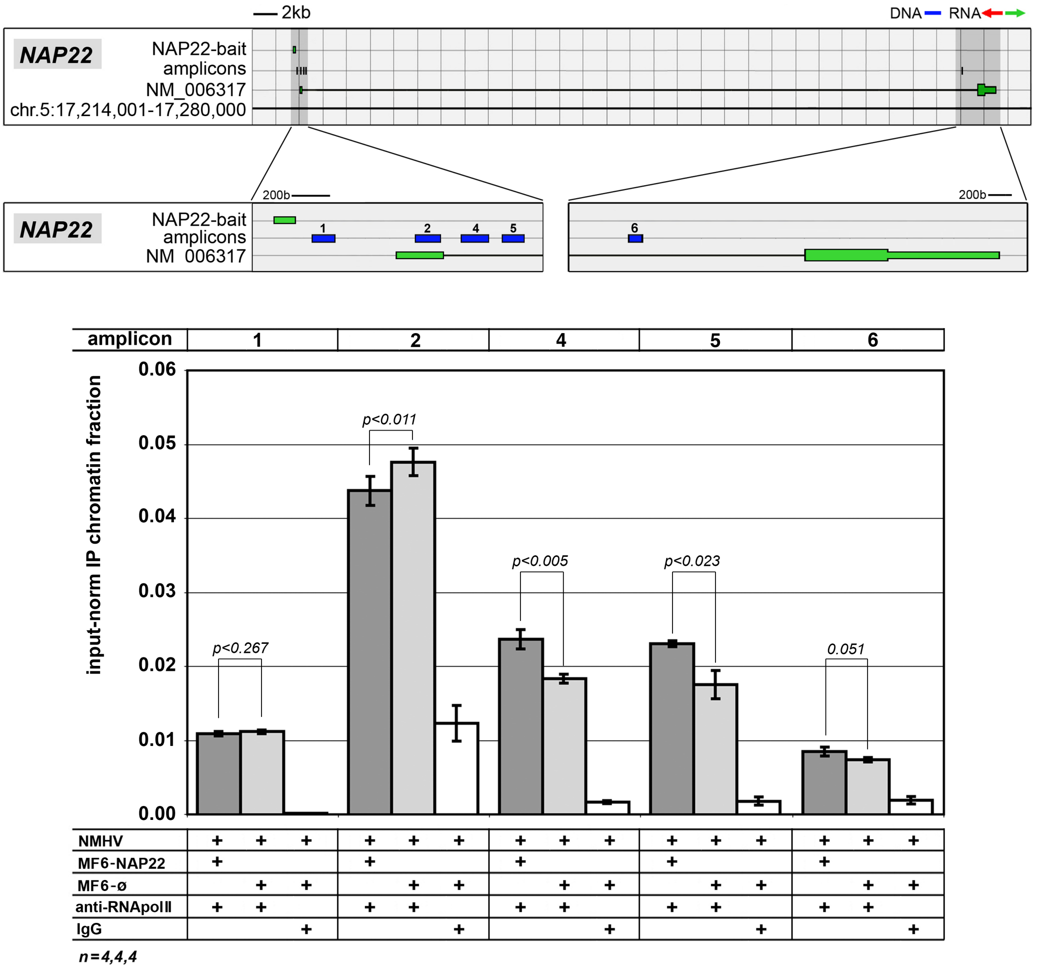

Supplementary Figure S7. Lentivirus dose-dependent infection of rhombo-spinal precursors. Correlation between multiplicity of infection (moi) and effective transduction rate, upon treatment of dissociated E10.5 rhombo-spinal precursors by the constitutive EGFP expressor pCCLsin.PPT.hPGK.EGFP.Wpre (37), as assessed 4 days upon infection. Tests performed in sextuplicate. Bars represent s.e.m.'s.

Figure S7

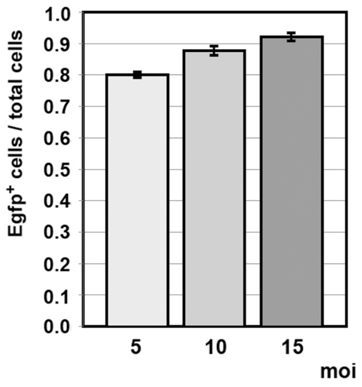

Supplementary Figure S8. Unaltered *Emx2* expression upon infection of cortico-cerebral precursors by control lentiviruses. *Emx2*-mRNA levels in uninfected cells and precursors co-infected with the control EGFP- and MF1-∅-encoding lentivectors. Results were normalized against *Gapdh* and further normalized against uninfected cells. Time-frame of the experiments as well as representation of moi's and statistical parameters were as in Figure 3

Figure S8

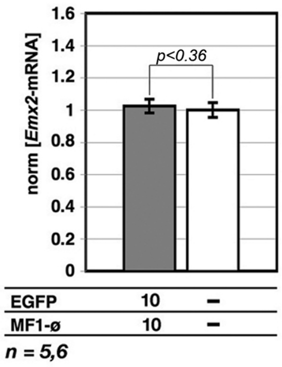

Supplementary Figure S9. Impact of *Emx2*-bait overexpression on neuronogenesis. No modulation of frequency of Tubb3[+] neurons in cultures of EGFP/MF1-Emx2-overexpressing precursors, as assayed by immunofluorescence. Results were normalized against EGFP/MF1-ø controls. Time-frame of the experiments as well as representation of moi's and statistical parameters were as in Figure 3.

Figure S9

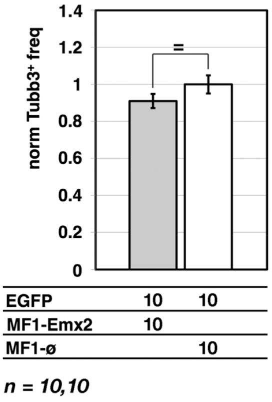

Supplementary Figure S10. Specificity of NMHV-mediated *Emx2* and *FoxG1* transactivation: no perturbation of other active genes and structurally similar, potential offtargets. Absence of aspecific gene transactivation in neural precursors overexpressing *Emx2* **(a)**, upon LV_NMHV / LV_MF2-Emx2 infection, or *FoxG1* **(b)**, upon LV_NMHV / LV_MF1-FoxG1 infection. *Pax6*, *Hes6*, *Sip1*, *Couptf1*, *Nf1a* and *Lhx2* are randomly chosen genes active in cortico-cerebral precursors. *Arid1a, CachD1, Ptpn12 and Dvl3* are potential offtargets, with 5' ends displaying remarkable homology to the *Emx2* bait (see Supplementary Table S6). Results were normalized against *Gapdh* and further normalized against EGFP/MF2-ø and EGFP/MF1-ø controls, respectively. Time-frame of the experiments as well as representation of moi's and statistical parameters were as in Figure 3.

Figure S10

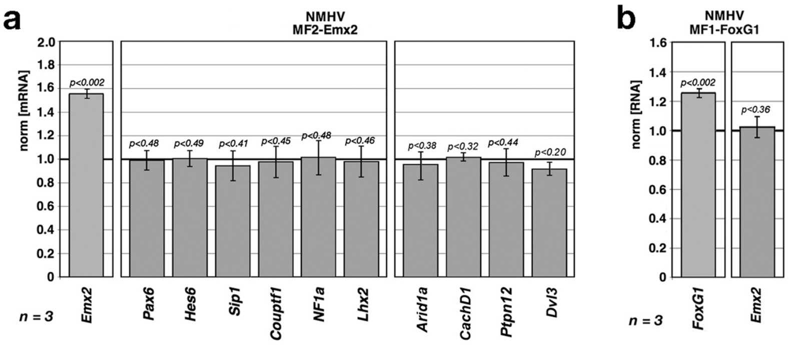

Supplementary Figure S11. Specificity of NMHV-mediated *Emx2* and *FoxG1* transactivation: limited cis-propagation and polarity effects. (a) No upregulation of *Emx2* mRNA, in cortical precursors co-expressing NMHV and the *antisense*-oriented, MF1-type, Emx2-AS bait. *Emx2* mRNA upregulation by NMHV and the corresponding sense-oriented bait is shown as a reference. **(b)** No upregulation of transcripts stemming from TSSs >1.5kb far from the bait-target, in neural precursors co-expressing NMHV and the MF1-type, *FoxG1* bait. Time-frame of the experiments, representation of moi's, data normalization and statistical parameters were as in Figure 3.

Figure S11

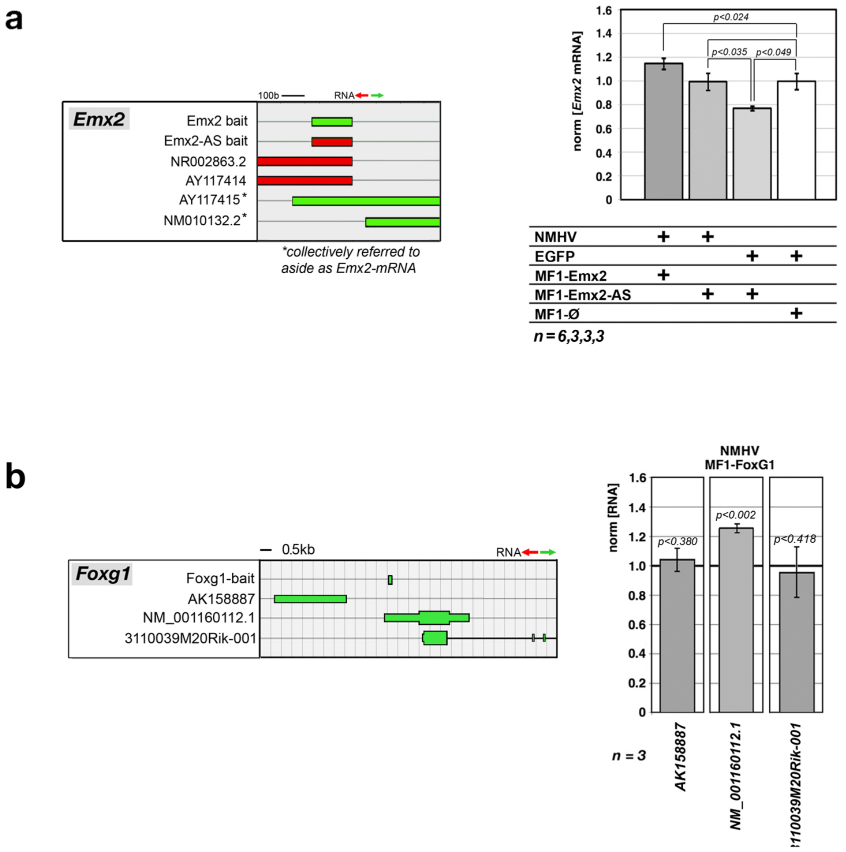

Supplementary Table S1. Wild type baits I

bait name	bait genomic coordinates	bait length	bait orient -ation vs TSS	bait midpoint distance from TSS	bait CG%	bait cloning oligos - sequence (5'-3')
FMR1.1	hg19_chrX(+)_nt 146,993,634-146,993,748	115b	S	+222b	68%	FMR1b/F - CCCGGATCCCCTCGAGCGCCCGCAGCCCA<br>FMR1b /R - CCCGTCGACCTTGTAGAAAGCGCCATTGGAGCCC
FMR1.2	hg19_chrX(+)_nt 146,993,101-146,993,220	120b	S	-308b	61%	FMR1-UPTSb.S/F - CCCGGATCCCCAGAGAGGCCGAACTGGGATAACC<br>FMR1- UPTSb.S/R - CCCGTCGACGCTGAAGGGCGGTGACAGGTCGCA
NAP22	hg19_chr5(+)_nt 17,217,026-17,217,142	117b	S	+153b	62%	NAP22b/F - CCCGGATCCCGTCGGGAGCGGGCTGGACTG<br>NAP22b/R - CCCGTCGACGTTGGATAAGAAGTGCTCGGGCTCTC
NRGN	hg19_chr11(+)_nt 124,609,848+124,609,995	148b	S	+92b	74%	NRGRb/F - CCCGGATCCCGAGAGCAGAGCTGCTGTTTCGGC<br>NRGRb/R - CCCCTCGAGGGTGCAGCAGTCCATGCTGGTGTC
FMR1.1-AS	hg19_chrX(-)_nt 146,993,634-146,993,748	115b	AS	+222b	68%	FMR1b-AS/F - CCCGGATCCCTTGTAGAAAGCGCCATTGGAGCCC<br>FMR1b-AS/R - CCCGTCGACCCTCGAGCGCCCGCAGCCCA
FMR1.2-AS	hg19_chrX(-)_nt 146,993,101-146,993,220	120b	AS	-308b	61%	FMR1-UPTSb.AS/F - CCCGGATCCGCTGAAGGGCGGTGACAGGTCGCA<br>FMR1- UPTSb.AS/R - CCCGTCGACCCAGAGAGGCCGAACTGGGATAACC
NAP22-AS	hg19_chr5(-)_nt 17,217,026-17,217,142	117b	AS	+153b	62%	NAP22b-AS/F - CCCGGATCCGTTGGATAAGAAGTGCTCGGGCTCTC<br>NAP22b-AS/R - CCCGTCGACCGTCGGGAGCGGGCTGGACTG
NRGN-AS	hg19_chr11(-)_nt 124,609,848+124,609,995	148b	AS	+92b	74%	NRGRb-AS/F - CCCGGATCCGGTGCAGCAGTCCATGCTGGTGTC<br>NRGRb-AS/R - CCCCTCGAGCGAGAGCAGAGCTGCTGTTTCGGC
FMR1.1.60L	hg19_chrX(+)_nt 146,993,634-146,993,698	65b	S	+197b	74%	FMR1b/F - CCCGGATCCCCTCGAGCGCCCGCAGCCCA<br>FMR65/R - CCCGTCGACTCTTCTCTTCAGCCCTGCTAGCGCC
FMR1.1.60R	hg19_chrX_(+)nt 146,993,688-146,993,748	61b	S	+249b	56%	FMR61/F - CCCGGATCCTGAAGAGAAGATGGAGGAGCTGGTGG<br>FMR1b /R - CCCGTCGACCTTGTAGAAAGCGCCATTGGAGCCC
NAP22.60L	hg19_chr5(+)_nt 17,217,026-17,217,090	65b	S	+126b	65%	NAP22b/F - CCCGGATCCCGTCGGGAGCGGGCTGGACTG<br>NAP65/R - CCCGTCGACGTGATGAATGACACCCCCTGGCACC
NAP22.60R	hg19_chr5(+)_nt 17,217,080-17,217,142	63b	S	+181b	54%	NAP63/F - CCCGGATCCTCATTCATCACCTCGAGGCAGCTCCCTA<br>NAP22b/R - CCCGTCGACGTTGGATAAGAAGTGCTCGGGCTCTC

Supplementary Table S2. Wild type baits II

bait name	bait genomic coordinates	bait length	bait orient -ation vs TSS	bait midpoint distance from TSS	bai t CG%	bait cloning oligos - sequence (5'-3')
Emx2 (Emx2.179)	mm10_chr19(+)_nt 59,458,457-59,458,635	179b	S	+174b	70%	Emx2.179-S/F - CCCAGATCTGCCGCAGGAGCGAGGGAGCTAGC<br>Emx2.179-S/R - CCCCTCGAGGACTCTTTAAAGTGCGTCAGAGGGAGG
Emx2.120	mm10_chr19(+)_nt 59,458,457-59,458,576	120b	S	+144b	74%	Emx2.179-S/F - CCCAGATCTGCCGCAGGAGCGAGGGAGCTAGC<br>E2b.120/R - GGGCTCGAGGGCCCAGAATGGAACGGAGTCGG
Emx2.60	mm10_chr19(+)_nt 59,458,457-59,458,516	60b	S	+114b	73%	Emx2.179-S/F - CCCAGATCTGCCGCAGGAGCGAGGGAGCTAGC<br>E2b.60/R - GGGCTCGAGCCGCCGACCTCTTGGTGTCTTCGCC
Emx2.30	mm10_chr19(+)_nt 59,458,457-59,458,486	30b	S	+99b	77%	E2b.30/F - GATCTGCCGCAGGAGCGAGGGAGCTAGCCCGGAGCC<br>E2b.30/R - TCGAGGCTCCGGGCTAGCTCCCTCGCTCCTGCGGCA
Emx2-AS	mm10_chr19(-)_nt 59,458,457-59,458,635	179b	AS	+174b	70%	Emx2.179-AS/F - CCCAGATCTGACTCTTTAAAGTGCGTCAGAGGGAGG<br>Emx2.179-AS/R - CCCCTCGAGGCCGCAGGAGCGAGGGAGCTAGC
FoxG1	mm10_chr12_nt 49,383,099-49,383,244	146b	S	+165b	59%	FoxG1.146-S/F - CCCAGATCTAGTACTGCCTAGAAGCTGAAGAGG<br>FoxG1.146-S/R - CCCCTCGAGACCCAAACAGTCCCGAAATAAAGC

Supplementary Table S3. qRTPCR oligos

gene	oligos - sequence (5'-3')
3110039M20Rik-001	RykM20.1/F - CCTGCCCTCCATACTCCAGCTTCTCC<br>RykM20.1/R - CCCGCACTGGGTGAGGCGATCTC
AK158887	AK158887.1108/F - TGGACTGCATTGATTCCTTGGGCAACTAGG<br>AK158887.1250/R - TTGAGCCCCTGGTAAGTAAGACACTCCAG
Arid1a	Arid1a.201/F - GCAGATGGAACACCCAAGACAGAATCCAAATC<br>Arid1a.201/R - TCTCGGGCTCACCACCCAACTCATACA
CachD1	Cachd1-001/F - GTGAACAGCCGAGCCTTCAACCCAGGA<br>Cachd1-001/R - CCCTTCTTCTGAGCTGAAATATTGCCACTTGAT
Couptf1	Couptf1/F - CTTCATGGACCACATCCGCATCTTTCAGGAACAG<br>Couptf1/ - TCACATACTCCTCCAGGGCACACTGTGATTTCTC
Dvl3	Dvl3.ex13/F - CTACATCTTTGGCGACCTCTGTGGTAACAT<br>Dvl3.ex14/R - GGAGCCAGTGTGTCCTGGTCAGAG
EGR3	EGR3.001/F - CGGCAGCAGCGACTCGGTAGTCCATT<br>EGR3.001/R - GCTGGAAGGAGCCGGAGTAAGAGAGTT
Emx2	Emx2.E2S/N2F - GGAAAGGAAGCAGCTGGCTCACAGTCTCAGTCTTAC<br>Emx2.E2S/N2R - GTGGTGTGTCCCTTTTTTCTTCTGTTGAGAATCTGAGCCTTC
ENST000000531241	ENST531241/F - ATTACCTTTGAAAATGCTCAGTTCTTCTTCGGTC<br>ENST531241/R - TCCTATCTCCTGTGTTTGAGTTCATCCTG
ENST00000456072	ENST456072/F - ATGCCTGCCTTGGTTATGGATGTGACAC<br>ENST56072/R - CTGATCGTAGCTCACTGTAGCCTCAAACTC
FMR1	hFMR1/F - GAAGATTCAATAACAGTTGCATTTGAAAACAACTGG<br>hFMR1/R - GCTCTGGAATACACCTCAACTTCATCAC
FMR-AS1-v1	NR024503/F - CCATTCCTGAAAAGCACTCAAACTGGACTTGAA<br>NR024503/R - GGGCTCCAATGGCGCTTTCTACAAGG
FoxG1	FoxG1.2193/F - TGGAGTGTCAGCGAGGTGCAATGTGG<br>FoxG1.2303/R - TACTGCACACATGGAAATCTGGCAGCC
FXR1	FXR1.001/F - GCGGCTCTAACGGGGCTTTCTACAAGG<br>FXR1.001/R - CTTCATTAAATGGAACCTGGCGTTCTGGTTG
Gapdh	Gapdh.5/F - ATCTTCTTGTGCAGTGCCAGCCTCGTC<br>Gapdh.5/R - GAACATGTAGACCATGTAGTTGAGGTCAATGAAGG
Hes6	Hes6/FI - GGAAGCACGGGGGGACCGCAAG<br>Hes6/RE - TGCACTGGATGTAGCCAGCAGCGAAG
Lhx2	Lhx2/ForM - GGCAAGCCCTTCCTCGGGGCAGCCGCATC<br>Lhx2/RevM - GCACACAACGCTGCCCGGTCACTGCTGATGGACGG
NM_006317 (NAP22-var1)	hNAP22/F - GGGGCTGCATAGGCACCCAGAG<br>hNAP22/R - CCTCGGCCTTCTTGTCTTTCTCCTTG
Nf1a	Nf1a/F - GGCCTTACTTCTCACACCCAGCCATCCGTTAC<br>Nf1a/R - GTGCACCTTGCCTTGACTGCTTCCATTGG
NM_001271606 (NAP22-var2)	Nap22/F2 - CTCACCCCAACCTCGCTGCCAG<br>Nap22/R2 - GGCCTTCTTGTCTTTCTCCTTGGCTTTC
NRGR	hNRGR/F2 - GGAGACTAGGCCAGAACTGAGCATTTTCAA<br>hNRGR/R2 - CAGGGAAGTCTCGTCGCTGCGAAG
Pax6	Pax6/ForM - CCAAGGGCGGTGAGCAGATGTGTGAGATCTTCTATTCTAG<br>Pax6/RevM - CCCGTTGACAAAGACACCACCAAGCTGATTCACTC
Ptpn12	Ptpn12.001/FN - GCCTTTGGCGAATACAGTCATAGACTTCTGGA<br>Ptpn12.001/R - CTTTTTCCTTCCCATCTCAAATTCTCGACAGGC
SCL4A2	SCL4A2/F - CTCCCTCTCCTTCCGCAGTCTCCT<br>SCL4A2/R - CAGAGATGACCATCTGCTCCACCAC
Sip1	Sip1/F2 - CGAGAGGCATATGGTGACGCACAAG<br>Sip1/R1 - CACTGTGAATTCTCAGGTGTTCTTTCAGGT

Supplementary Table S4. ChIP oligos

ChIP amplicon	oligos - sequences (5'-3')
NAP22_amplicon 1	NapIP/F - GAGAAGTCCACGGACAGCCCAGG<br>NapIP/R - GTGCCCCGCCAACGTTTCCTAAATG
*NAP22*_amplicon 2	Nap225UTR/F - GCTCCAACTGGCTCCTCGCTCC<br>Nap225UTR/R - GTGCCTATGCAGCCCCTGAGTTAG
*NAP22*_amplicon 3	Nap22jun/F - GGGGCTGCATAGGCACCCAGAG<br>Nap22jun/R - CTGCTGCCCGCTGCATGAAGGGA
*NAP22*_amplicon 4	Nap22i1/F - TCCCTTCATGCAGCGGGCAGCAG<br>Nap22i1/R - CCCTCCACCATGCGAGCGGC
*NAP22*_amplicon 5	Nap22i3/F - GGTGACGGGAGGAATTGCCGAATG<br>Nap22i3/R - CTTTCTCCCAGGCTGGTAAGGGCT
*NAP22*_amplicon 6	Nap22i2/F - CAGTTCTGCCAGTGGACCTGACCTG<br>Nap22i2/R - GTCCCATAAATGAGAAAAGCCCAGGAG
SLC4A2	SLC4IP/F2 - GCTATTGGTCACACTGGCCCAGAGG<br>SLC4IP/R2 - CTGAGGCCGCAGGAGGAAGTCCAT
Emx2	Emx2.α/Fint - ATTTACCAGGCTTCGGAGGAAGCTG<br>Emx2.α/Rint - CATGGAAGTGTTCGCTTCTTCACTAGTA
CachD1	CachdIP/F - AAAGTTTGGCGGGCTGCGCCTCC<br>CachdIP/R - CCGGCCTTACGGTCTCCTCTTCCT
Dvl3	Dvl3IP/F - CCGTACCTGGTGAAGCTGCCCCT<br>Dvl3IP/R - CCAAAATCGTCGTCCATAGACTTGAAGAAG
Ptpn12	PtpnIP/F - GGGACTTCATGGTGAGTGTCCGTTCT<br>PtpnIP/R - CCTGTCCTCGCACCGCACACAAA

Supplementary Table S5. Off-target/on-target comparisons

<table>
<tr><th>off-target gene (on-target gene)</th><th>off-target sequence location:
- hg19 coordinates
- distance from off-target gene TSS, relative orientation</th><th>off-target vs on-target comparison</th><th>gap density</th><th>homology</th></tr>
<tr><td>*SLC4A2*
(*NAP22*)</td><td>- chr.7(-):150759353-150759431
- circa -0.3kb, antisense</td><td>off 1 GGGTCTGAGACGGGTTGCTGGGTGAGAGACCCA-GGGTTCTCAAAGTTCCTCAACCGCTG 59
|||||||||| |||| ||| |||| ||| ||| | ||| ||| || ||| | |
on 22 GGGTCTGAGAAGGGTAGCT-GGTG------CCAGGGGGTGTCA---TTCATC-ACCTC-G 69

off 60 AGGCTAAGCTACCTAGGTCT 79
|||| |||| |||| ||||
on 70 AGGC--AGCTCCCTAAGTCT 87</td><td>15/80
(19%)</td><td>57/80
(71%)</td></tr>
<tr><td>*FXR1*
(*FMR1*)</td><td>- chr.3(+):180,630,471-180,630,524
- circa +0.3kb, sense</td><td>off 1 AACATGGCGGAGCTGACGGTGGAGGTTCGCGGCTCTAACGGGGCTTTCTACAAG 54
|| |||| ||||||| |||||| || || ||||| || || ||||||||||||
on 62 AAGATGGAGGAGCTGGTGGTGGAAGTGCGGGGCTCCAATGGCGCTTTCTACAAG 115</td><td>0/54
(0%)</td><td>44/54
(81%)</td></tr>
<tr><td>*EGR3*
(*NRGN*)</td><td>- chr.8(+):22,550,683-22,550,780
- circa +0.05kb, sense</td><td>off 5 GGGTGCCTCAGCTGGTGCGGTATGAGGCTGGGTCGT-GGG-GGGGGTGGGGCGTGTGCGG 62
|||| |||| || || || | ||||||| || ||| |||||||||| |||
on 121 GGGT-CCTCTGCGGGGACG--A--AGGCTGG--CGCAGGGCGGGGGTGGGGTCTGT---- 73

off 63 GGGGTG--GGGTGGGGGCTGGGCTGGGG 88
| |||| ||||||||||| ||||||
on 72 GTGGTGCCGGGTGGGGGCT---CTGGGG 48</td><td>18/88
(20%)</td><td>62/88
(70%)</td></tr>
<tr><td></td><td></td><td>off 59 GCGGGGGGTGGGGTGGGGGCTGG-GCTGGG-GGGGGATCT 96
|| ||||||||||| | | ||| || ||| ||||| |||
on 89 GC-GGGGGTGGGGTCTGTG-TGGTGCCGGGTGGGGGCTCT 52</td><td>4/40
(10%)</td><td>31/40
(78%)</td></tr>
<tr><td></td><td></td><td>off 31 GCTGG-GTCGTGGGGGGGGTGGGGCGTGTGCGGGG-GGTGGGGTGGGGGCTGGCTGGGG 88
||||| |||| |||||| | | ||||||| | || | || || |||| ||||
on 133 GCTGGTGTCG----GGGGGT----CCTCTGCGGGGACGAAGGCT-GGCGCAGGGCGGGGG 83

off 89 GGGGATCT 96
 ||| |||
on 82 TGGGGTCT 75</td><td>11/68
(16%)</td><td>46/68
(68%)</td></tr>
</table>

Supplementary Table S6. Off-target/on-target comparisons

<table>
<tr><th>off-target gene (on-target gene)</th><th>off-target sequence location:
- mm10 coordinates
- distance from off-target gene TSS, relative orientation</th><th>off-target vs on-target comparison</th><th>gap density</th><th>homology</th></tr>
<tr><td>Arid1a
(Emx2)</td><td>- chr.4(+):133754037-133754110
- -circa 0.4kb, antisense</td><td>off   4 AGGAGGGAGGGAGC--G---GGGGGGGAGGGGG--GAC-CCGGGACG--GGCGGGCGGGC 53
         ||||| |||||||| | || | | |||| | ||| || || | ||||| |||||
on   6 AGGAGCGAGGGAGCTAGCCCGGAGCGCAGGGCGAAGACACCAAGAGGTCGGCGGCCGGGC 65

off 54 GGGCGGGCTGGAGG 67
         |||| |||||||||
on   66 GGGC-GGCTGGAGG 78</td><td>11/74
(15%)</td><td>54/74
(73%)</td></tr>
<tr><td>Cachd1
(Emx2)</td><td>- chr.4(+):100776869-100776966
- circa +0.2kb, antisense</td><td>off   5 GGAAAGCGAAGGCGGCGC--C-GCTCCGCCGGTGCTCTC-AGCCGCCCGCCCGGCCTGCG 60
         |||| || || |||||| | || ||| | |||| ||| |||||||||||||||| ||
on 110 GGAA--CGGAGTCGGCGCTACTGCCCCGGCTGTGC-CTCCAGCCGCCCGCCCGGCC-GC- 56

off 61 GGCG-CCGC--G--GTCTTCGAGCCGCTGC-CGCCGGGC 93
           || || | | |||||| ||| |||| | ||||||
on   55 --CGACCTCTTGGTGTCTTC--GCC-CTGCGCTCCGGGC 22</td><td>20/99
(20%)</td><td>72/99
(73%)</td></tr>
<tr><td>Ptpn12
(Emx2)</td><td>- chr.5(+):21,055,747-21,055,837
- circa +0.01kb, sense</td><td>off   5 CCCG-C-GTCCCCCCCGCCGCCCGCCCGCGCGCCGCAGCGCCCGCT--CCGTCTTC-CCC 59
         |||| | || || || |||||||||||| ||||| || || || |||||| |||
on   88 CCCGGCTGTGCCTCCAGCCGCCCGCCCG---GCCGC--CGACCTCTTGGTGTCTTCGCCC 34

off 60 AGCTCGCTCCGGCC---CGCCCTCCCTCC 85
          | |||||||| | | ||||| ||||
on   33 TG--CGCTCCGGGCTAGCTCCCTCGCTCC 7</td><td>15/89
(17%)</td><td>63/89
(71%)</td></tr>
<tr><td>Dvl3
(Emx2)</td><td>- chr.16(+):20,517,023 -20,517,140
- circa +0.15kb, sense</td><td>off 6 GCGGGCGGCGCTGGGACCCGG-GAGC-GGCCGGAGA-AC-AAGGGAGCT-GGCGGCCCGG 60
        ||| | || ||| | ||||| | || || || ||| || ||| || | ||||||| ||
on 10 GCGAG-GGAGCTAG--CCCGGAGCGCAGGGCGAAGACACCAAG--AGGTCGGCGGCCGGG 64

off 61 CCGGCGGCTGAGTGGGGCTGAGCAGCGGGCCGCGCCG-AGCGCCGAGCTC 109
        | |||||||| | ||| | |||| || | || | |||||||| |||
on 65 CGGGCGGCTG---GAGGC--A-CAGCCGG--G-GCAGTAGCGCCGA-CTC 104</td><td>21/110
(19%)</td><td>77/110
(70%)</td></tr>
</table>

Supplementary Table S7. Mutant, NAP22.60L-derivative baits.

bait	*sequence (5' → 3')*
NAP22.60L	CGTCGGGAGCGGGCTGGACTGGGGTCTGAGAAGGGTAGCTGGTGCCAGGGGGTGTCATTCATCAC
NAP22.60L.mut30.1	CGTCGGGAGCGGGCTGGACTGGGACTCAGAGGAAACGATCAACGCCAGGGGGTGTCATTCATCAC
NAP22.60L.mut30.2	CGTCGGGAGCGGGCTAAGTCAAAACCTGAGAAGGGTAGCTAACATTGAAAGGTGTCATTCATCAC
NAP22.60L.mut30.3	CGTCGGGAGCGAATCAAGCTGGGGTCTGAAGGAAACAGCTGGTGCCAAAAAACATCATTCATCAC
NAP22.60L.mut30.4	CGTCGGGAGTAAATTGGACTGGGACTCAAGAAGGGTAATCAATGCCAGGGGACACTATTCATCAC
NAP22.60L.mut15.1	CGTCGGGAGCGGGCTGGACTGGGGTCTCTCTTCCCATGCTGGTGCCAGGGGGTGTCATTCATCAC
NAP22.60L.mut15.2	CGTCGGGAGCGGGCTGGAGACCCGTCTGAGAAGGGTAGCTGGACGGTGGGGGTGTCATTCATCAC
NAP22.60L.mut15.3	CGTCGGGAGCGGGGACGACTGGGGTCTGAGTTCCGTAGCTGGTGCCAGCCCGTGTCATTCATCAC
NAP22.60L.mut15.4	CGTCGGGAGCGCCGTGGACTGGGGTGAGAGAAGGGTAGGAGGTGCCAGGGGCACTCATTCATCAC
NAP22.60L.mut15.5	CGTCGGGAGCCCGCTGGACTGCCGTCTGAGAACCGTAGCTGGTCGCAGGGGGTGAGATTCATCAC
NAP22.60L.mut15.6	CGTCGCGAGCGCGCTGGTCTGGGCTCTGACAAGGGAAGCTGCTGCCACGGGGTCTCATTGATCAC
